# Chemical Investigation of the Global Regulator *veA*-Overexpressed Mutant of an Arctic Strain *Aspergillus sydowii* MNP-2

**DOI:** 10.3390/md24010034

**Published:** 2026-01-09

**Authors:** Qing Gong, Wei Wang, Yujie Zhao, Xiaoying Wang, Xuelian Bai, Huawei Zhang

**Affiliations:** 1 School of Pharmaceutical Sciences, Zhejiang University of Technology, Hangzhou 310014, China; 221124070243@zjut.edu.cn (Q.G.); w923748440@163.com (W.W.); zhao_yujie010@163.com (Y.Z.); 211123070050@zjut.edu.cn (X.W.); 2 State Key Laboratory of Green Chemical Synthesis and Conversion, Zhejiang University of Technology, Hangzhou 310014, China; 3 College of Life and Environmental Sciences, Hangzhou Normal University, Hangzhou 311121, China

**Keywords:** *Aspergillus*, global transcription regulator, secondary metabolites, biosynthetic gene cluster, activation

## Abstract

A growing body of evidence indicates that artificial manipulation of transcriptional regulation is a powerful approach to activate cryptic biosynthetic gene clusters (BGCs) of secondary metabolites (SMs) in fungi. In this study, one mutant strain MNP-2-OE::*veA* was constructed by overexpressing the global transcription regulator *veA* in an Arctic-derived strain *Aspergillus sydowii* MNP-2. Chemical investigation of the mutant OE::*veA* resulted in the isolation of one novel polyhydroxy anthraquinone (**1**) together with nine known metabolites (**2**–**10**), which were unambiguously characterized by various spectroscopic methods including 1D and 2D NMR and HR-ESI-MS as well as via comparison with literature data. Biosynthetically, compounds **1** and **10** as new arising chemicals were, respectively, formed by type II polyketide synthase (T2PK) and non-ribosomal peptide synthetase (NRPS), which were silent in the wild-type (WT) strain MNP-2. A bioassay showed that only compound **3** had weak inhibitory effect on human pathogen *Candida albicans,* with a MIC value of 64 ug/mL, and **4** displayed in vitro weak cytotoxic activity against HCT116 cells (IC_50_ = 44.47 μM). These results indicate that overexpression of *veA* effectively awakened the cryptic BGCs in fungal strains and enhanced their structural diversity in natural products.

## 1. Introduction

Marine-derived *Aspergillus* fungi produce a diverse array of structurally unique and biologically active secondary metabolites (SMs), representing a hotspot in current field of natural product research [1,2,3,4,5]. *A. sydowii*, a ubiquitous fungus widely distributed across various ecosystems, is renowned for its extraordinary environmental adaptability and impressive secondary metabolic potential [6]. Genome sequencing and bioinformatics analyses suggest that the number of SM biosynthetic gene clusters (BGCs) in *A. sydowii* strains far exceeds the chemical diversity of compounds produced by them [7,8]. Therefore, the vast majority of these BGCs remain transcriptionally silent, making the discovery of novel structures challenging. In the past two decades, multiple strategies, such as epigenetic regulation, promoter engineering, global regulation, and others, have been developed and employed to activate silenced BGCs, thereby activating fungal secondary metabolic capabilities and discovering diversified SMs [9,10,11]. Of these approaches, global regulatory factor modification is considered one of the most promising strategies due to its ability to simultaneously affect multiple BGCs and its straightforward operability. *veA* is one of the most prevalent global regulators within the *Aspergillus* genus, playing a pivotal role in modulating morphogenesis and secondary metabolism [12,13,14].

**Figure 1:**
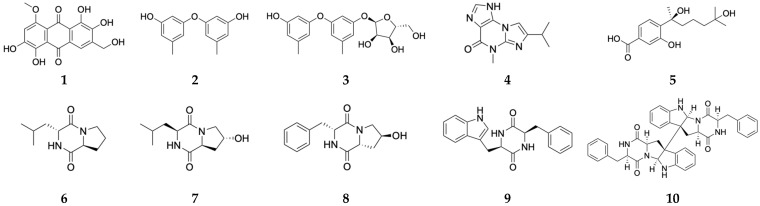
Chemical structures of compounds (**1**–**10**) from the mutant OE::*veA*.

In our previous studies, we performed whole-genome analysis and metabolic profiling of an Arctic-derived fungus *A. sydowii* MNP-2 using the global natural product social molecular network (GNPS) approach, suggesting that this strain harbors biosynthetic potential to produce diverse SMs with complex structures and significant biological activities [15]. The chemical study of the wild-type (WT) strain MNP-2 had afforded 11 phenolic bisabolane sesquiterpenes (PBSs) from its rice and potato dextrose broth (PDB) fermentation extracts [16]. To awaken the latent BGCs in this strain to produce other bioactive SMs, one global regulator *veA*-overexpressed mutant OE::*veA* was successfully constructed and shown to make ten substances (**1**–**10**, Figure 1) including one novel polyhydroxy anthraquinone (**1**). This work highlights the construction of the *veA*-overexpressed mutant OE::*veA* and the isolation and elucidation of its derived SMs, as well as their biological properties.

## 2. Results and Discussion

### 2.1. Construction and Morphological Characterization of the Mutant Strain

**Figure 2:**
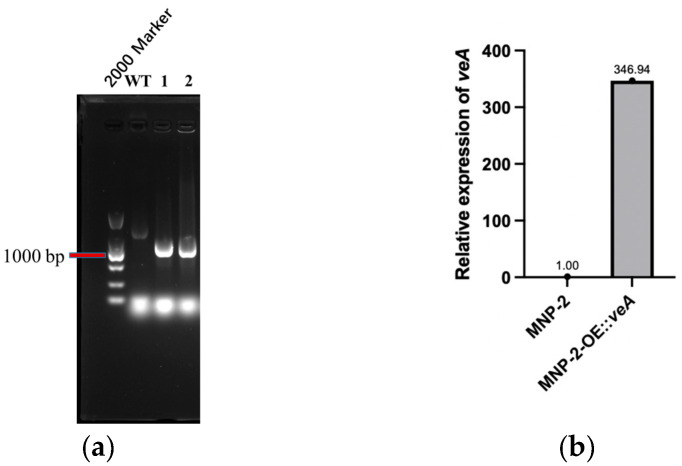
Verification of *veA*-overexpressing mutant. (**a**) Amplification results of the anti-hygromycin fragment of *veA*-overexpressed transformants. Lane 1 and 2: OE::*veA*. (**b**) The relative expression level of the *veA* gene was verified by RT-qPCR.

Following genome sequencing and gene annotation analysis, the global regulator *veA* from *A. sydowii* MNP-2 (1755 bp in full length) was identified and selected as the target of this study. Specific primers were employed for the amplification of the *veA* gene, and the overexpression plasmid pOE::*veA* was subsequently generated (Figures S1 and S2). The overexpression mutant OE::*veA* was constructed using *Agrobacterium tumefaciens*-mediated transformation (ATMT) [17,18]. Furthermore, the *Hyg* resistance gene band could be specifically amplified from the transformed strains harboring the recombinant plasmid (Figure 2a). Real-time quantitative polymerase chain reaction (RT-qPCR) was performed to determine the expression level of *veA* in the OE::*veA* strain, with *β-actin* serving as the internal reference. Compared with the WT MNP-2, the *veA* gene expression level in the OE::*veA* strain was significantly increased by 346.94-fold (Table 1 and Figure 2b). Collectively, these findings confirmed the successful construction of the *veA*-overexpressing mutant OE::*veA*.

**Figure 3:**
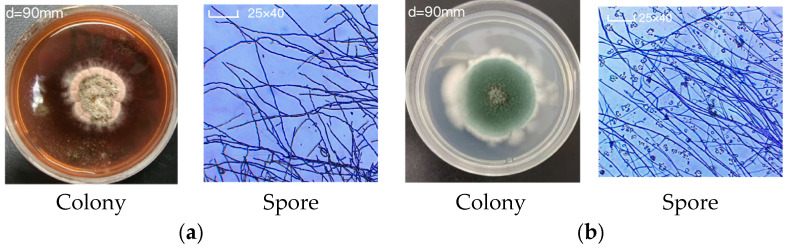
Colony and spore morphology of strains MNP-2 (**a**) and OE::veA (**b**) on PDA.

The WT strain *A. sydowii* MNP-2 was observed to produce significantly higher levels of red pigment, whereas the *veA*-overexpressing mutant OE::*veA* exhibited a marked reduction in pigment biosynthesis. Compared to the WT strain, OE::*veA* displayed three distinct colony phenotypic alterations: more pigmented spores, reduced surface wrinkling, and significantly larger colony diameters (Figure 3a). At the microscopic level, the WT strain MNP-2 formed dense, complex mycelial networks characterized by fewer conidia and minimal branching at hyphal tips. In contrast, OE::*veA* produced an increased number of conidia and developed longer, dendritic hyphal branches (Figure 3b).

### 2.2. LC-MS/MS Analysis of Mutant-Derived Crude Extracts

**Figure 4:**
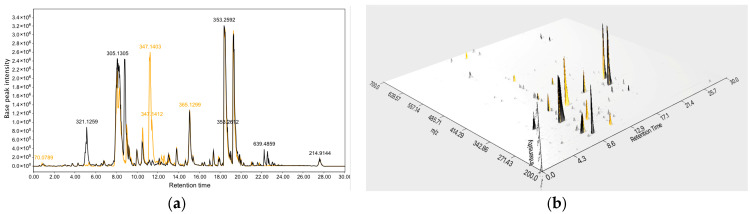
LC-MS/MS analysis of crude extracts of strains MNP-2 (yellow) and OE::*VeA* (black). (**a**): Total ion chromatogram. (**b**): Three-dimensional visualization spectra.

The liquid chromatography–mass spectrometry (LC-MS) data are shown in Figure 4. When cultured in modified Martin medium, the molecular weights of SMs from the WT strain MNP-2 were primarily concentrated with in the *m/z* 250–500 range, whereas those produced by the mutant strain OE::*veA* were concentrated in the *m/z* 200–400 range.

### 2.3. Isolation and Identification of OE::veA-Derived Secondary Metabolites

A total of ten compounds (**1**–**10**) were isolated from the modified Martin medium culture extract, comprising one novel compound (**1**) and nine known metabolites (2–10), including a rare diphenyl ether glycoside (3). Using HR-ESIMS and ^1^H NMR as well as comparison with literature data, these compounds were, respectively, identified as follows: 1,2,5,6-tetrahydroxy-3-(hydroxymethyl)-8-methoxy-9,10-anthraquinon (1) [19], diorcinol (2) [20], diorcinol-3-O-α-d-ribofuranoside (3) [21], acremolin B (4) [22], 10-hydroxysydonic acid (5) [23], cyclo (Leu-Pro) (6) [24], cyclo (*L*-Phe-4-OH-*L*-Pro) (7) [25], cyclo (*L*-(4-OH)-Pro-*L*-Leu) (8) [26], cyclo (*L*-Phenyl-*L*-tryptophyl) (9) [27], and WIN 64821 (10) [28].

**Figure 5:**
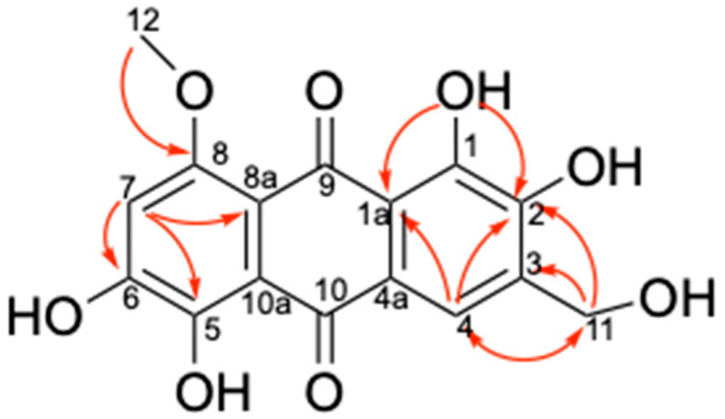
The key HMBC correlation of compound **1**.

Compound **1**, obtained as a deep red powder that easily dissolves in DMSO-*d_6_*, is slightly soluble in methanol, and insoluble in chloroform. The molecular formula of 1 was established as C_16_H_12_O_8_ based on the sodium adduct molecular ion peak m/z 355.0433 [M+Na]^+^ obtained from HR-ESIMS, indicating a degree of unsaturation of 11. The ^1^H NMR spectrum (Figure S3) of 1 exhibits a methyl signal at *δ*_H_ 3.90 (3H, *s*), a methylene signal at *δ*_H_ 4.58 (2H, *s*), two aromatic protons at *δ*_H_ 6.99 (1H, *s*) and *δ*_H_ 7.88 (1H, *s*), and five active hydrogen signals at *δ*_H_ 13.97 (1H, *brs*), *δ*_H_ 13.87 (1H, *brs*), *δ*_H_ 11.28 (1H, *brs*), *δ*_H_ 10.51 (1H, *brs*), and *δ*_H_ 5.37 (1H, *brs*) (Table 2). Further analysis of its ^13^C NMR spectrum (Figure S4) indicates that compound **1** contains two ketone carbonyl signals at *δ*_C_ 185.4 and 187.2, along with twelve sp^2^-hybridized carbon signals at *δ*_C_ 157.1, 155.5, 149.4, 149.3, 147.6, 134.5, 121.9, 117.6, 115.6, 114.9, 108.7, and 106.3 ppm, which are similar to those of asperthecin, an anthraquinone pigment obtained from *Aspergillus nidulans* [19]. The key HMBC correlations from H-11 (*δ*_H_ 4.58) to C-2 (*δ*_C_ 149.4), C-3 (*δ*_C_ 134.5), and C-4 (*δ*_C_ 117.6), the oxygen methyl (*δ*_H_ 3.90) to C-8 (*δ*_C_ 157.1) and from H-7 (*δ*_H_ 6.99) to C-5 (*δ*_C_ 147.6), C-6 (*δ*_C_ 155.5), and C-8a (*δ*_C_ 108.7) support the assignment of the hydroxymethyl at the C3 position and the methoxy substitution at the C8 position (Figure 5). Therefore, compound **1** was unambiguously determined as 8-methoxyl-asperthecin.

Compound **2**: Light yellow oily substance; soluble in methanol and chloroform. It is commonly distributed in soil fungi (serving as defensive metabolites against pathogens). Previous studies have documented its antimicrobial activity against Gram-positive bacteria *Staphylococcus aureus*, *Bacillus subtilis,* and the yeast *Candida albicans* [20].

Compound **3**: Colorless oily substance; soluble in methanol and chloroform. Initially isolated from marine microbes, it exhibits cytotoxicity against multiple cancer cell lines, including human tissue cell lymphoma, human prostate cancer, and human colorectal adenocarcinoma cell lines [21].

Compound **4**: White crystals; soluble in methanol and chloroform. It is primarily distributed in deep-sea-derived fungi [22].

Compound **5**: White amorphous powder; soluble in methanol and chloroform. First isolated from marine fungi, it shows obvious inhibitory activities against pathogenic bacteria *Escherichia coli*, *Edwardsiella tarda*, *Vibrio harveyi*, and *Vibrio parahaemolyticus* [23].

Compounds **6**–**8**: Colorless crystals; soluble in methanol and chloroform. Originally isolated from deep-sea sediments, prior bioinformatic analysis indicated that their biosynthesis is closely associated with four genes (*jatA–D*), which encode core non-ribosomal peptide synthetases and acetyltransferases [24,25,26].

Compound **9**: White amorphous powder; soluble in methanol and chloroform. It was previously isolated from an unidentified *Penicillium* sp. [27].

Compound **10**: White amorphous powder; soluble in methanol, slightly soluble in chloroform. It was initially derived from *Aspergillus* sp. It acts as a substance P (SP)-competitive antagonist binding to the human NK1 receptor, and also possesses significant NK2 receptor antagonistic activity [28].

### 2.4. Transcriptomic Analysis

**Figure 6:**
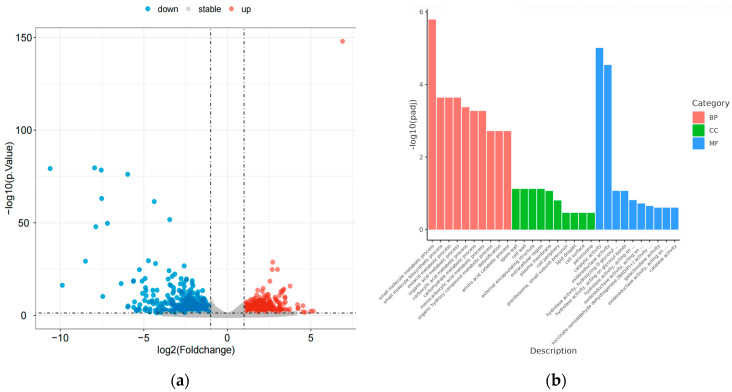
Differential gene expression between the wild type and OE::*veA*.((**a**) the volcano plots of DEGs; (**b**) gene ontology enrichment analysis column chart of DEGs).

To investigate the role of global regulatory factor *veA* in the biosynthesis of natural products in the mutant OE::*veA*, differential expression gene (DEG) analysis was carried out using transcriptomics technology. The results suggested that the mutant OE::*veA* had 608 upregulated genes and 635 downregulated genes compared with the WT strain MNP-2. Volcano plot analysis further validated the statistical significance of their gene expression differences (Figure 6a). Gene ontology (GO) enrichment analysis indicated that overexpression of the *veA* gene significantly affected metabolic process-related pathways in the strain, suggesting that this gene may be involved in regulating natural product biosynthesis through the modulation of metabolic networks (Figure 6b).

**Figure 7:**
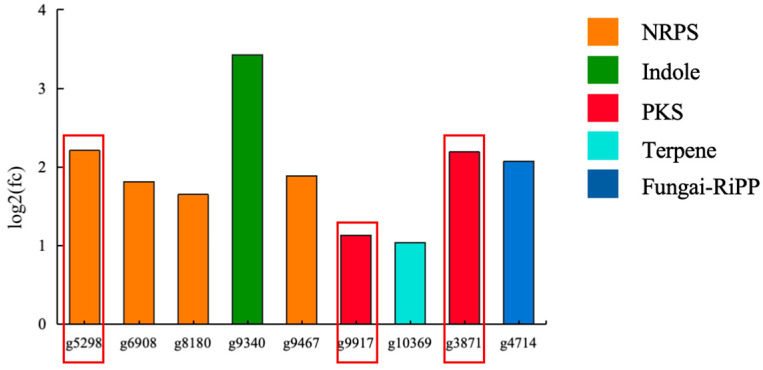
OE::*veA* significantly upregulates core biosynthetic genes.

Among these greatly upregulated (*p* < 0.05) genes in the mutant OE::*veA*, nine SM biosynthesis-related genes were identified and shown to encode two polyketide synthases (PKSs), four non-ribosomal peptide synthetases (NRPSs), one terpene, one ribosomally synthesized and post-translationally modified peptide (RiPP), and one indole (Figure 7). Biosynthetically, compounds **1** and **10**, as new arising SMs from the mutant OE::*veA,* were presumably formed by T2PK and NRPS, respectively. By Blastp-based homology alignment analysis, two upregulated PKS genes (g3871 and g9917) had a respective similarity of 56.6% and 46.43% with *mdpG,* which was essential for the biosynthesis of T2PK natural product xanthones [29]. The core gene (g5298), which exhibited upregulated expression in NRPS, shared 67% sequence similarity with *dtpA*, a key gene within the BGC responsible for the production of TDKPs [30]. Therefore, the overexpression of the *veA* gene activated the PKS and NRPS genes in the mutant OE::*veA* and resulted in the production of compounds **1** and **10**.

### 2.5. Biological Activity of SMs

Antimicrobial assay revealed that compound **3** showed weak activity against *C. albicans* (MIC = 64 μg/mL). The cytotoxicity results for compounds (**1**–**10**) indicate that compound **4** with an IC_50_ value of 44.47 μM against HCT116 cells.

## 3. Materials and Methods

### 3.1. Fungal Material and Fermentation

The fungal strain *A. sydowii* MNP-2 was isolated from Arctic marine sediments. In this study, an overexpression strain of the global regulator *veA*, designated, was constructed from the WT MNP-2. For seed culture, OE::*veA* was inoculated into the modified Martin medium and incubated in the dark at 28 °C with shaking at 180 rpm for 15 days. For hyphal observation, 1 μL of a spore suspension (1 × 10^7^ spores/mL) from both the WT and OE:: *veA* strains was aseptically placed into the gap between a PDA plate and a sterile coverslip. The plates were incubated at 28 °C in the dark for 2 days. The coverslips were then carefully removed with sterile forceps, placed onto glass slides, and stained with crystal violet. Hyphal growth and morphology were examined under an optical microscope.

### 3.2. Construction of Overexpression Mutant

Using the newly extracted genomic cDNA of *A. sydowii* MNP-2 as a template, primer pairs containing the target gene were designed for *veA* gene amplification. Following the PCR amplification of the target gene fragment, the *veA* gene was cloned downstream of the gpdA promoter in the fungal expression vector via restriction enzyme digestion and ligation, constructing the recombinant plasmid pOE::*veA*. The validated overexpression vector was introduced into *Agrobacterium tumefaciens* AGL-1 via freeze–thaw transformation. The successful transformant colonies were isolated and co-cultured with *A. sydowii* MNP-2. After the resistance screening, overexpressing positive transformants were obtained. RNA was extracted from positive transformants, reverse transcribed into cDNA, and validated via RT-qPCR. After ten generations of stable passage on antibiotic plates, RNA was extracted from overexpressing mutant strains and WT strains, was reverse transcribed into cDNA templates, and subjected to RT-qPCR to detect *veA* gene expression levels in the WT and mutant OE::*veA* strains. In the RT-qPCR analysis for assessing the expression level of the *veA* gene in the OE::*veA* mutant and WT MNP-2 strains, *β-actin* was utilized as the internal reference gene. Specifically, the ΔCt value for each sample was calculated as the difference between the cycle threshold (Ct) of the target gene (*veA*) and that of the internal reference gene (β-actin). Subsequently, the WT MNP-2 strain served as the control: its average ΔCt value was first determined, and the ΔΔCt value was then derived by subtracting this average ΔCt value from the ΔCt value of the OE::*veA* mutant. Finally, the relative overexpression level of *veA* in the OE::*veA* mutant was calculated using the 2^−ΔΔCt^method [31], which directly reflects the fold increase in *veA* expression in the mutant strain relative to the WT counterpart.

Using sterile pipettes, spore suspensions (spore concentration 1 × 10^7^ spores/mL) of the WT and mutant strains were inoculated onto PDA plates. Plates were incubated at 28 °C for 8 days under dark conditions, with daily growth records taken for both strains. The positive mutant strain OE::*veA* maintained normal growth after the tenth generation, exhibiting significant phenotypic differences compared to the MNP-2 strain. The primers used in this study are detailed in Table S1.

### 3.3. General Experimental Procedures

^1^H NMR and ^13^C NMR spectra were recorded at 600 and 150 MHz, respectively, using Bruker Avance DRX600 instruments (Bruker, Fällande, Switzerland). ESIMS were obtainedwith an Agilent 6210 LC/TOF-MS spectrometer (Agilent Technologies, Santa Clara, CA, USA). Reverse phase HPLC wascarried out on an Essentia LC-16P apparatus (Essentia, San Diego, CA, USA) fitted with apreparative HPLC column (Phenomenex Gemini-NX C18 column 50 mm × 21.2 mm, 5 μm; Phenomenex, Inc., Torrance, CA, USA). It operated at a flow rate of 1.0 mL/min. All the chemicals used were of analytical grade.

### 3.4. Liquid Chromatography–Mass Spectrometry Analysis

A small amount of fermented crude extract was dried by blowing air, and then was adjusted to a concentration of 1 mg/mL. Centrifugation was performed at 12,000 rpm and 4 °C for 20 min, and the supernatant was collected into a sample vial. The instrumentation used was a SCIEX X500B quadrupole time-of-flight (QTOF) high-resolution mass spectrometer (HRMS) coupled with a SCIEX ExionLC ultra-high-performance liquid chromatograph (UPLC; SCIEX, Framingham, MA, USA). The liquid chromatography conditions were as follows: the analytical column was a Phenomenex Kinetex C18 column (100 Å, 150 × 2.1 mm, 5 μm; Phenomenex, Inc., Torrance, CA, USA), with gradient elution performed using a methanol/water (MeOH/H_2_O) mobile phase system ranging from 10% to 100% MeOH at a constant flow rate of 0.3 mL/min. Mass spectrometry (MS) conditions were configured as follows: electrospray ionization (ESI) was employed as the ion source, with full-scan mode performed for both positive and negative ion modes. The mass-to-charge ratio (*m/z*) range was set at 100–1500 for both full MS scan and MS/MS fragmentation analysis.

### 3.5. Extraction and Isolation

About 10.9 g of crude extract obtained from the fermentation broth of mutant strain OE::*veA* cultivated in modified Martin medium using ethyl acetate as extraction solvent was subjected to semi-preparative HPLC (Gemini NX-C18 110Å, 50 × 21.2 mm, 5 μm; Phenomenex, Inc., Torrance, CA, USA) using an acetonitrile–water gradient elution (30:70 to 100:0, *v*/*v*), yielding seven fractions (Fr.1–Fr.7). Fr.3 was further purified by gel column chromatography with a mobile phase of methanol/dichloromethane (1:1, *v*/*v*), affording four subfractions (Fr.3.1–Fr.3.4). Fr.3.2 was subsequently purified by semi-preparative HPLC (Gemini C18 110Å, 250 × 4.6 mm, 5 μm; Phenomenex, Inc., Torrance, CA, USA) using acetonitrile/water (15:85, *v*/*v*) as the mobile phase, yielding compounds **6** (5.4 mg, t_R_ = 23.0 min), **7** (8.0 mg, t_R_ = 11.2 min), and **8** (7.8 mg, t_R_ = 17.9 min). Fr.4 was subjected to semi-preparative HPLC (Gemini NX-C18 110Å, 250 × 21.2 mm, 5 μm; Phenomenex, Inc., Torrance, CA, USA) using an acetonitrile–water gradient elution (20:80 to 100:0, *v*/*v*), yielding seven subfractions (Fr.4.1–Fr.4.7). A red solid precipitated upon the vacuum concentration of Fr.4.2, which was isolated as compound **1** (11.2 mg) after filtration. Fr.4.3 was further purified via semi-preparative HPLC (Gemini C18 110Å, 250 × 4.6 mm, 5 μm; Phenomenex, Inc., Torrance, CA, USA) using acetonitrile/0.1% aqueous formic acid (30:70, *v*/*v*) as the mobile phase, producing compound **5** (7.2 mg, t_R_ = 9.7 min). Fr.4.4 was purified by semi-preparative HPLC (Gemini C18 110Å, 250 × 4.6 mm, 5 μm; Phenomenex, Inc., Torrance, CA, USA) to yield compounds **2** (7.9 mg, t_R_ = 17.5 min, acetonitrile/water, 42:58, *v*/*v*) and **10** (19.1 mg, t_R_ = 14.0 min, acetonitrile/0.1% aqueous formic acid, 45:55, *v*/*v*). Fr.4.5 was further subjected to purification using semi-preparative HPLC (Gemini C18 110Å, 250 × 4.6 mm, 5 μm; Phenomenex, Inc., Torrance, CA, USA) with a mobile phase of acetonitrile/water, yielding compounds **3** (3.1 mg, t_R_ = 23.0 min, 28:82, *v*/*v*), **4** (5.8 mg, t_R_ = 16.0 min, acetonitrile/water, 18:82, *v*/*v*), and **9** (7.4 mg, t_R_ = 43.2 min, acetonitrile/water, 22:78, *v*/*v*).

### 3.6. Transcriptomic Analysis

For each sample, three sets of biological replicates were prepared. Library construction and Illumina sequencing were performed at Shanghai Personal Biotechnology Co. Ltd. (Hangzhou, China). The genes of differential expression were analyzed by DESeq (v1.38.3) with screen conditions as follows: expression difference multiple |log2FoldChange| > 1, significant *p*-value < 0.05. TopGO (v2.50.0) was used to perform GO enrichment analysis on the differentially expressed genes (all DEGs/up DEGs/down DEGs), to calculate the *p*-value using the hypergeometric distribution method (the standard of significant enrichment is *p*-value < 0.05), and to identify the GO terms with significantly enriched differential genes to determine the main biological functions performed by these differential genes. Sequence similarity was analyzed using the Blastp algorithm against the NCBI Non-Redundant Protein Database with an E-value cutoff set at 1 × 10^−5^.

### 3.7. Bioactivity Assay

According to the gradient dilution method [32], the antimicrobial activity of compounds **1**–**10** was tested against three indicator strains, including *Staphylococcus aureus* (*S. aureus* ATCC 25923), *Escherichia coli* (*E. coli* ATCC 25922), and *Candida albicans* (*C. albicans* ATCC 10231). Three pathogenic strains were obtained from Nanjing Medical University (Nanjing, China). A crude extract solution at a concentration of 10 mg/mL was prepared using DMSO. Ampicillin sodium served as the positive control for *E. coli* ATCC 25922 and *S. aureus* ATCC 25923, while amphotericin B was used as the positive control for *C. albicans* ATCC 10231. DMSO was used as the negative control.

The human breast cancer cell line MCF-7, the human hepatocellular carcinoma cell line HepG2, and the human colorectal cancer cell line HCT116 were purchased from the China Center for Type Culture Collection (CCTCC, Wuhan, China). The cytotoxicity of all chemicals 1–10 against MCF-7, HepG2, and HCT116 tumor cells was evaluated using the CCK-8 assay [33]. MCF-7 and HepG2 cells were cultured in DMEM medium, while HCT116 cells were maintained in RPMI 1640 medium (supplemented with 10% fetal bovine serum and 1% penicillin-streptomycin). Cells were seeded into 96-well plates at a density of 5 × 10^3^ cells/well (100 μL/well) and incubated at 37 °C with 5% CO_2_ for 24 h. Different concentrations of compounds **1**–**10** were then added, followed by a further incubation for 48 h. After the 48 h incubation, a medium containing 10% CCK-8 was added to each well, and the plates were incubated for an additional 40 min. The optical density (OD) values were measured at a wavelength of 450 nm using a microplate reader.

## 4. Conclusions

One *veA*-overexpressing mutant MNP-2-OE::*veA* was successfully constructed and shown to make one novel polyhydroxy anthraquinone (**1**) together with nine known metabolites (**2**–**10**), which compounds **1** and **10**, respectively, biosynthesized by T2PK and NRPS, were newly emerging substances. These results demonstrated the crucial role of *veA* as a global transcription regulatory factor in activating silent BGCs in fungi, providing experimental evidence and theoretical support for enhancing chemical diversity through target gene regulation. In future, other transcription regulatory factors (such as *LaeA* and *VelB*) in the genome of WT strain MNP-2 will be further overexpressed or knocked out for discovery of novel SMs with therapeutic potential.

## Figures and Tables

**Table 1:** The real-time fluorescent quantitative PCR analysis results of *veA* gene.

Name	Sample CT Medium	Control	Control CT Medium	ΔCt	ΔΔCt
MNP-2	29.6012	*β* *-actin*	21.9699	7.63	/
OE::*veA*	22.5559	*β* *-actin*	23.3631	−0.81	−8.44

**Table 2:** ^1^H-NMR (600 MHz) and ^13^C-NMR (150 MHz) spectral data of compound **1** (*δ* in ppm, *J* in Hz).

Position	*δ*_H_ (*J* in Hz)	*δ*_C_	Position	*δ*_H_ (*J* in Hz)	*δ*_C_
1	-	149.3	1a	-	115.6
2	-	149.4	4a	-	114.9
3	-	134.5	8a	-	108.7
4	7.88 (1H, *s*)	117.6	10a	-	121.9
5	-	147.6	8-OCH_3_	3.90 (3H, *s*)	56.1
6	-	155.5	1-OH	13.97 (1H, *brs*)	-
7	6.99 (1H, *s*)	106.3	2-OH	11.28 (1H, *brs*)	-
8	-	157.1	5-OH	13.87 (1H, *brs*)	-
9	-	185.4	6-OH	10.51 (1H, *brs*)	-
10	-	187.2	11-OH	5.37 (1H, *brs*)	-
11	4.58 (2H, *s*)	57.6	4a	-	114.9

Recorded in DMSO-*d*_6._

## Data Availability

The original contributions presented in this study are included in the article. Further inquiries can be directed to the corresponding author(s).

## Supplementary Materials

The following supporting information can be downloaded at https://www.mdpi.com/article/10.3390/md24010034/s1. Figure S1: Plasmid map of pOE::*veA*. Figure S2: The sequencing results of pOE::*veA*. Figures S3–S10: 1D, 2D NMR, and HRESIMS spectra of 1,2,5,6-tetrahydroxy-3-(hydroxymethyl)-8-methoxy-9,10-anthraquinon (**1**). Figures S11 and S12: 1D NMR spectra and HRESIMS spectra of diorcinol (**2**). Figures S13 and S14: 1D NMR spectra and HRESIMS spectra of diorcinol-3-O-α-d-ribofuranoside (**3**). Figures S15 and S16: 1D NMR spectra and HRESIMS spectra of acremolin B (**4**). Figures S17 and S18: 1D NMR spectra and HRESIMS spectra of 10-hydroxysydonic acid (**5**). Figures S19 and S20: 1D NMR spectra and HRESIMS spectra of cyclo (Leu-Pro) (**6**). Figures S21 and S22: 1D NMR spectra and HRESIMS spectra of cyclo (L-Phe-4-OH-L-Pro) (**7**). Figures S23 and S24: 1D NMR spectra and HRESIMS spectra of cyclo (L-(4-OH)-Pro-L-Leu) (**8**). Figures S25 and S26: 1D NMR spectra and HRESIMS spectra of cyclo (L-Phenyl-L-tryptophyl) (**9**). Figures S27–S29: 1D NMR spectra and HRESIMS spectra of WIN 64821 (**10**). Table S1: List of all used primers and their sequence. Table S2: The NMR spectroscopic data for diorcinol (**2**). Table S3: The NMR spectroscopic data for diorcinol-3-O-α-d-ribofuranoside (**3**). Table S4: The NMR spectroscopic data for acremolin B (**4**). Table S5: The NMR spectroscopic data for 10-hydroxysydonic acid (**5**). Table S6: The NMR spectroscopic data for cyclo (Leu-Pro) (**6**). Table S7: The NMR spectroscopic data for cyclo (L-Phe-4-OH-L-Pro) (**7**). Table S8: The NMR spectroscopic data for cyclo (L-(4-OH)-Pro-L-Leu) (**8**). Table S9: The NMR spectroscopic data for cyclo (L-Phenyl-L-tryptophyl) (**9**). Table S10: The NMR spectroscopic data for WIN 64821 (**10**).

## Abbreviations

The following abbreviations are used in this manuscript:

RT-qPCR	real-time quantitative polymerase chain reaction
T2PK	type II polyketide
GNPS	global natural product social molecular network
NRPS	Non-ribosomal peptide synthetase
PBSs	phenolic bisabolane sesquiterpenes
BGCs	biosynthetic gene clusters
PDA	potato dextrose agar medium
PDB	potato dextrose broth medium
MIC	minimum inhibitory concentration
DEG	differential expression gene
SMs	secondary metabolites
GO	gene ontology
OD	optical density
