# Supplementary material for: Chemical Investigation of the Global Regulator veA-Overexpressed Mutant of an Arctic Strain Aspergillus sydowii MNP-2
Source: Mar Drugs. 2026 Jan 9;24(1):34. doi: 10.3390/md24010034 (PMC12843210; doi:10.3390/md24010034)
Supplement: Supplementary file 1 [file marinedrugs-24-00034-s001.zip › marinedrugs-4062455-supplementary.pdf]

Contents

Table S1. List of all used primers and their sequence....3
Figure S1. Plasmid map of pOE::*veA*.... **Error! Bookmark not defined.**
Figure S2. The sequencing results of pOE::*veA*....3
Figure S3. ^{1}H-NMR spectrum of compound **1** in DMSO-d_6 (600 MHz). 4
Figure S4. ^{13}C-NMR spectrum of compound **1** in DMSO-d_6 (150 MHz)....4
Figure S5. ESI-MS spectrum of compound **1**....5
Figure S6. DEPT-135 spectrum of compound **1** in DMSO-d_6....5
Figure S7. ^{1}H-^{1}H COSY spectrum of compound **1** in DMSO-d_6....6
Figure S8. HSQC spectrum of compound **1** in DMSO-d_6. 6
Figure S9. HMBC spectrum of compound **1** in DMSO-d_6. 7
Figure S10. NOESY spectrum of compound **1** in DMSO-d_6. 7
Figure S11. ^{1}H-NMR spectrum of compound **2** in DMSO-d_6 (600 MHz). 8
Table S2. The ^{1}H-NMR spectroscopic data for compound **2** in DMSO-d_6 (600 MHz). 8
Figure S12. ESI-MS spectrum of compound **2**....9
Figure S13. ^{1}H-NMR spectrum of compound **3** in DMSO-d_6 (600 MHz). 9
Table S3. The ^{1}H-NMR spectroscopic data for compound **3** in DMSO-d_6 (600 MHz). 10
Figure S14. ESI-MS spectrum of compound **3**....10
Figure S15. ^{1}H-NMR spectrum of compound **4** in DMSO-d_6 (600 MHz). 11
Table S4. The ^{1}H-NMR spectroscopic data for compound **4** in DMSO-d_6 (600 MHz). 11
Figure S16. ESI-MS spectrum of compound **4**....12
Figure S17. ^{1}H-NMR spectrum of compound **5** in DMSO-d_6 (600 MHz). 12
Table S5. The ^{1}H-NMR spectroscopic data for compound **5** in DMSO-d_6 (600 MHz). 12
Figure S18. ESI-MS spectrum of compound **5**....14
Figure S19. ^{1}H-NMR spectrum of compound **6** in DMSO-d_6 (600 MHz). 14
Table S6. The ^{1}H-NMR spectroscopic data for compound **6** in DMSO-d_6 (600 MHz). 15
Figure S20. ESI-MS spectrum of compound **6**....15
Figure S21. ^{1}H-NMR spectrum of compound **7** in DMSO-d_6 (600 MHz). 16
Table S7. The ^{1}H-NMR spectroscopic data for compound **7** in DMSO-d_6 (600 MHz). 16
Figure S22. ESI-MS spectrum of compound **7**....17
Figure S23. ^{1}H-NMR spectrum of compound **8** in CD_3OD (600 MHz)....17
Table S8. The ^{1}H-NMR spectroscopic data for compound **8** in DMSO-d_6 (600 MHz). 18
Figure S24. ESI-MS spectrum of compound **8**....18
Figure S25. ^{1}H-NMR spectrum of compound **9** in DMSO-d_6 (600 MHz). 19
Table S9.The ^{1}H-NMR spectroscopic data for compound **9** in DMSO-d_6 (600 MHz). 19
Figure S26. ESI-MS spectrum of compound **9**....20
Figure S27. ^{1}H-NMR spectrum of compound **10** in DMSO-d_6 (600 MHz)....20
Table S10. The ^{1}H-NMR spectroscopic data for compound **10** in DMSO-d_6 (600 MHz). ...21
Figure S28. ^{13}C-NMR spectrum of compound **10** in DMSO-d_6 (150 MHz)....22
Figure S29. ESI-MS spectrum of compound **10**....22

Table S1. List of all used primers and their sequence.

Name	Sequence (5'-3')
veA-F	GGTAGATCTGACTAGATGGCTGCGAGAGCTCC
veA-R	ATTCGAGCTGGTCACTTAATGCATGGCAGGAGAGATCTTCG
pveA-F	AAATATCGTGCCTCTCCTGCTT
pveA-R	GTCGATATCTCACGGGAGGAAC
Hyg-F	CTATTTCTTTGCCCTC
Hyg-R	TGAAAAAGCCTGAACT
tin-F	GTCTCCATGAAGGAGGTTGAG
tin-R	GAAGGTGGAGGACATCTTGAG
qveA-F	ATGGCTGCGAGCTC
qveA-R	TTAATGCATGGCAGGAGAGAT

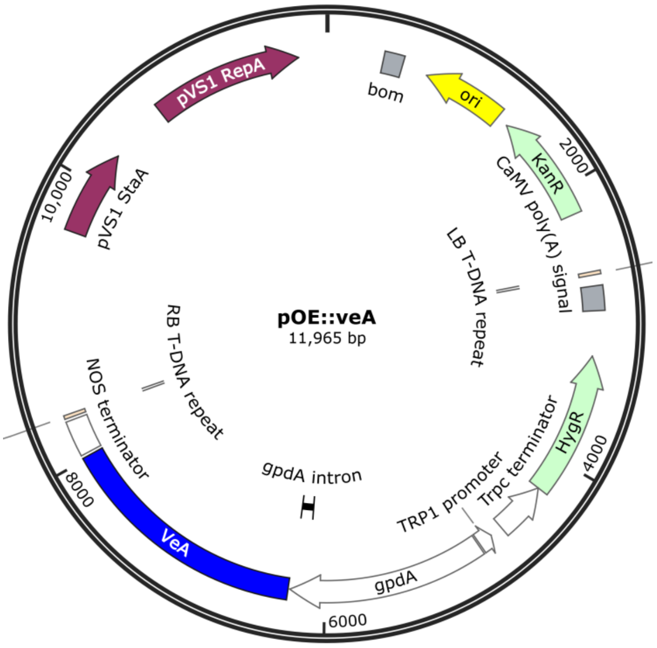

Figure S1. Plasmid map of pOE::*veA*.

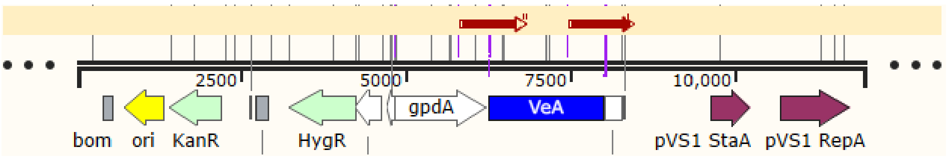

Figure S2. The sequencing results of pOE::*veA*

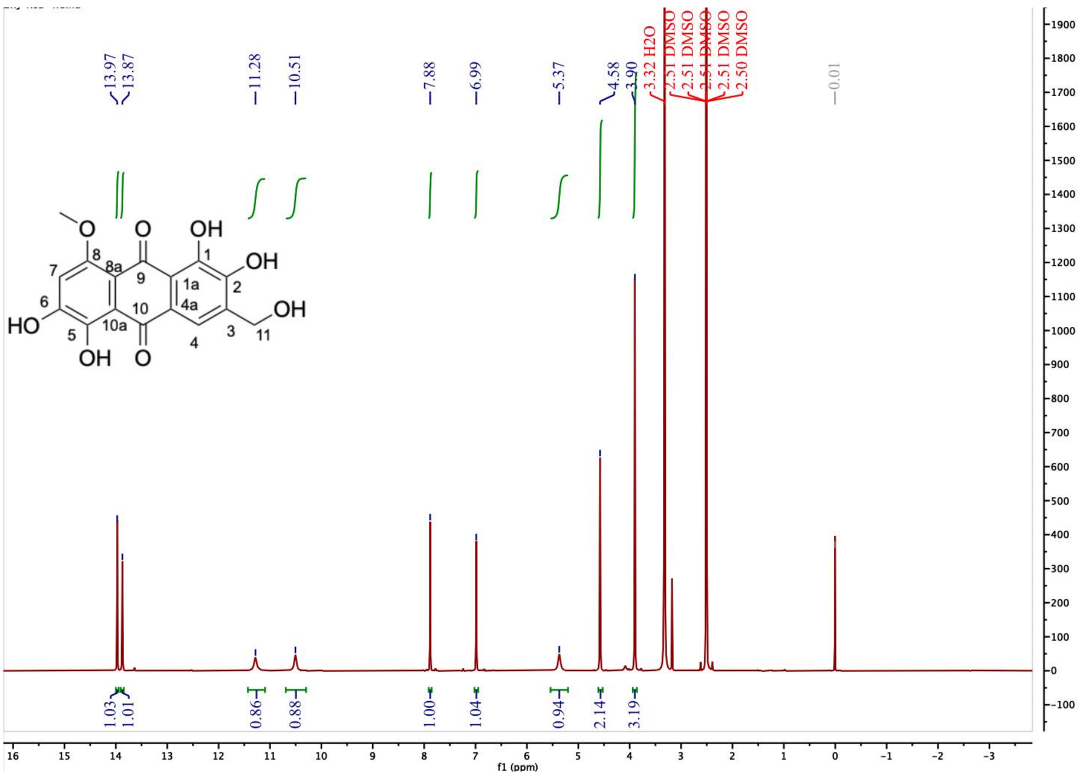

Figure S3. ^{1}H-NMR spectrum of compound **1** in DMSO-*d*$_6$ (600 MHz).

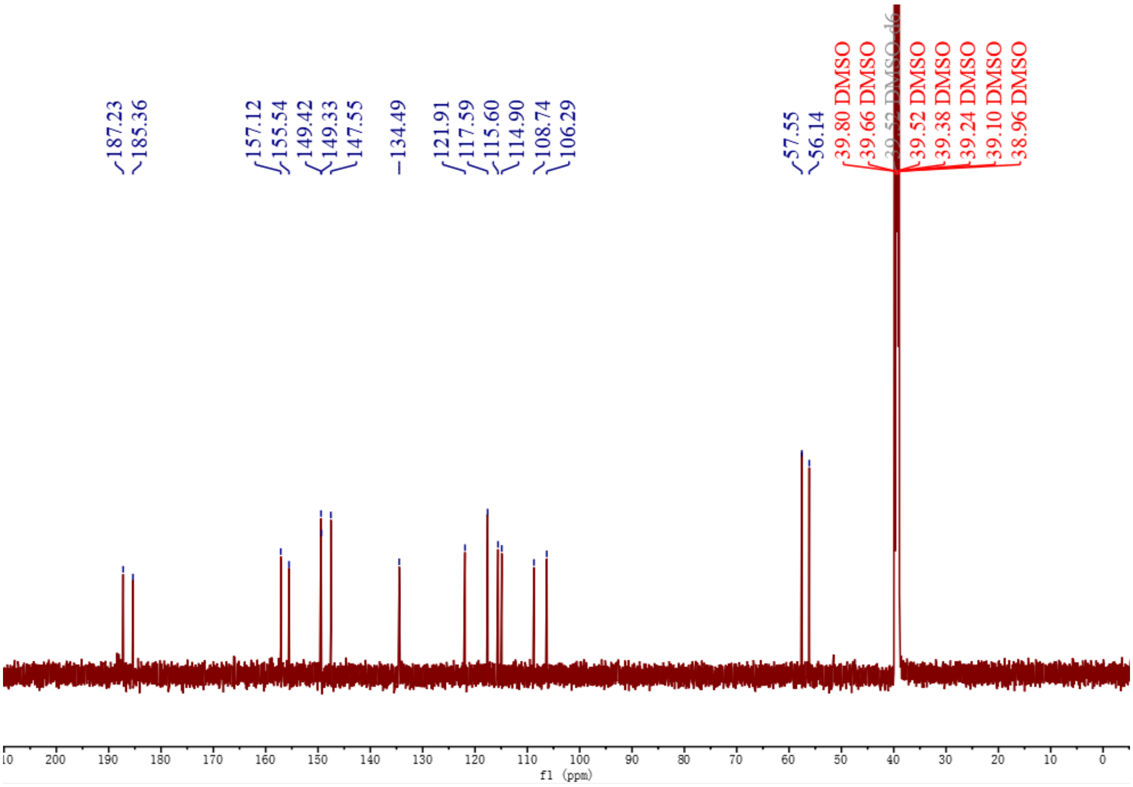

Figure S4. ^{13}C-NMR spectrum of compound **1** in DMSO-*d*$_6$ (150 MHz).

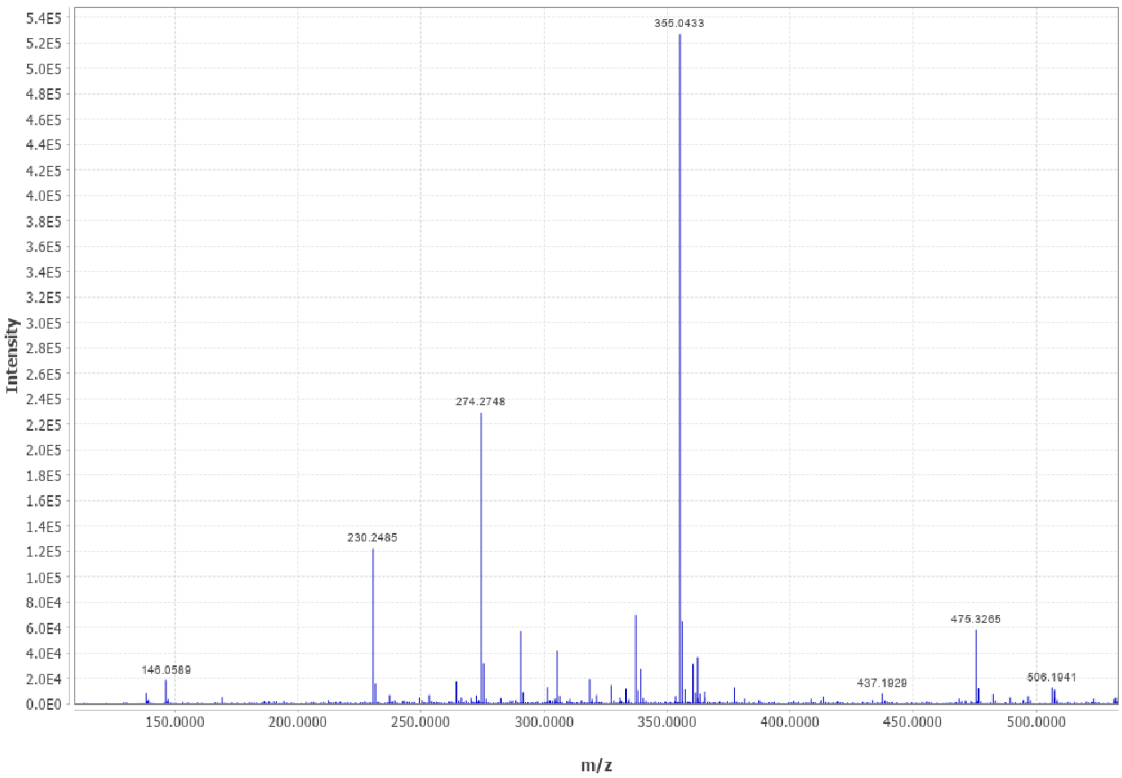

Figure S5. ESI-MS spectrum of compound **1.**

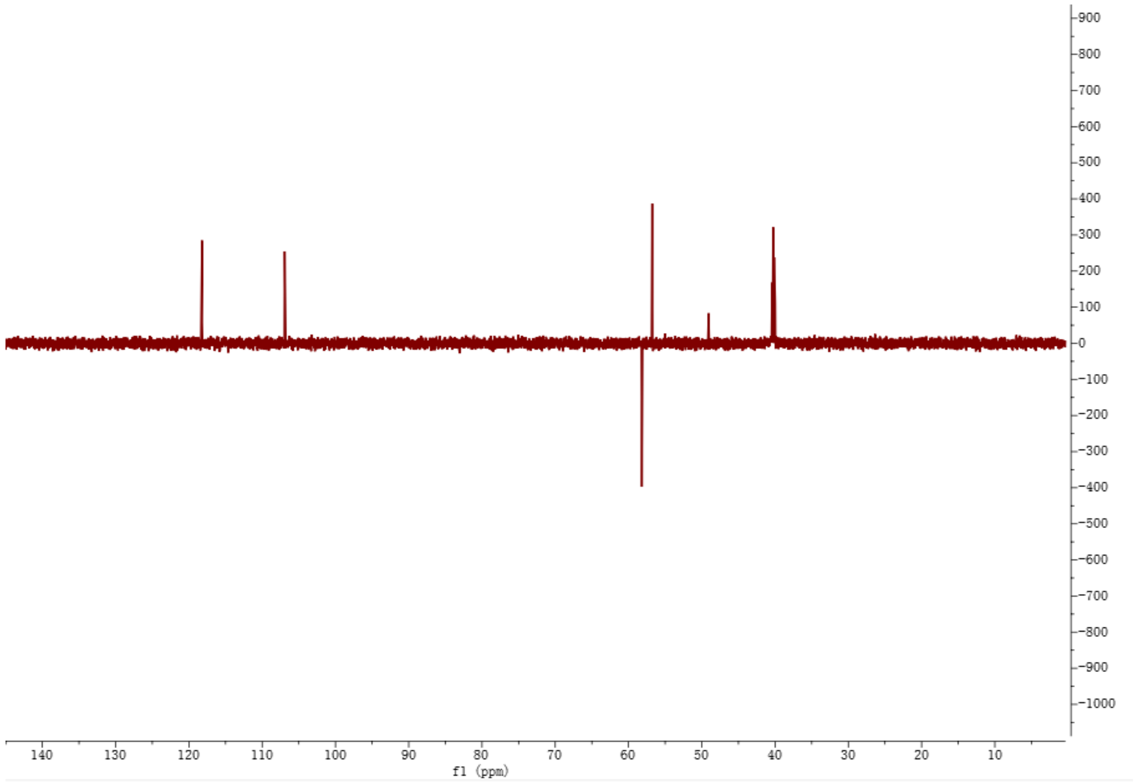

Figure S6. DEPT-135 spectrum of compound **1** in DMSO-d_6.

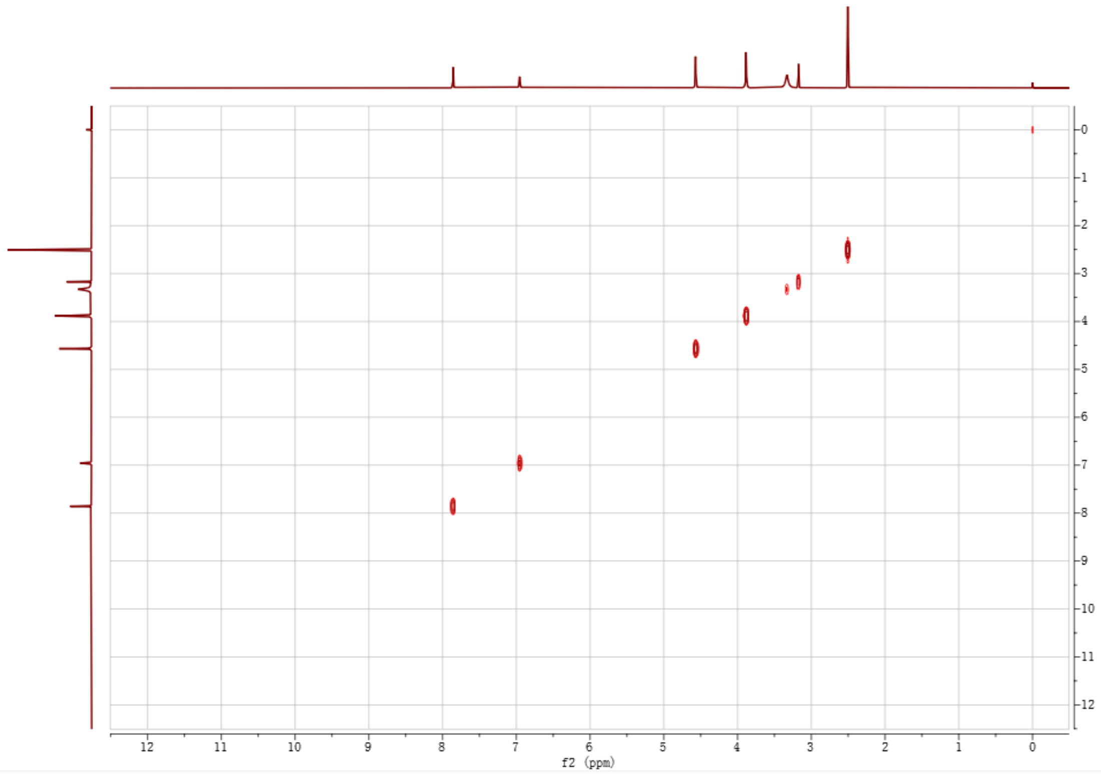

Figure S7. ^{1}H-^{1}H COSY spectrum of compound **1** in DMSO-d_6.

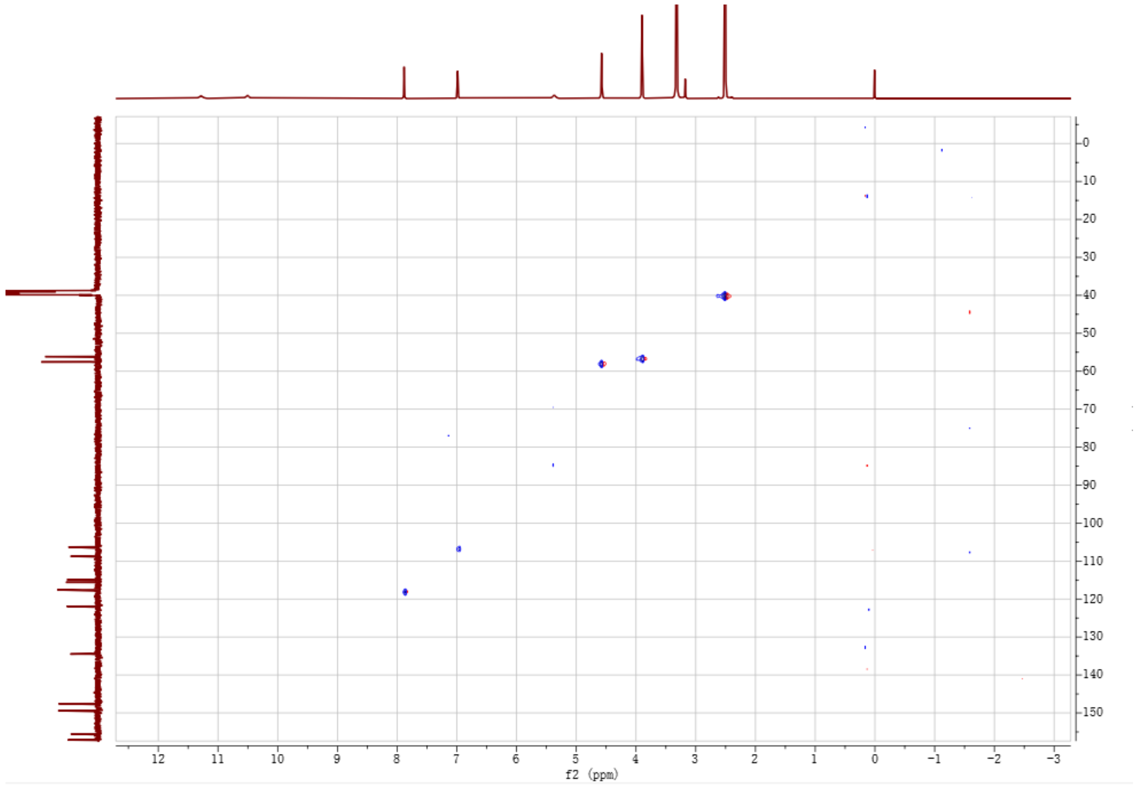

Figure S8. HSQC spectrum of compound **1** in DMSO-d_6.

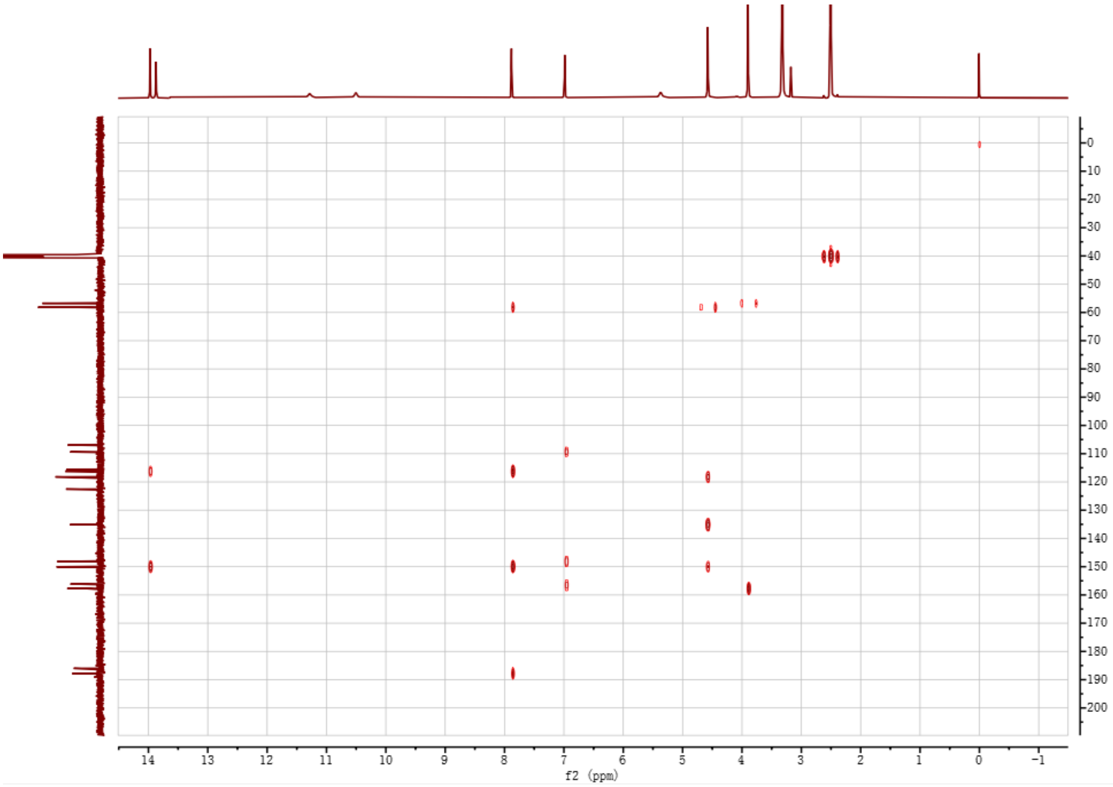

Figure S9. HMBC spectrum of compound **1** in DMSO-d_6.

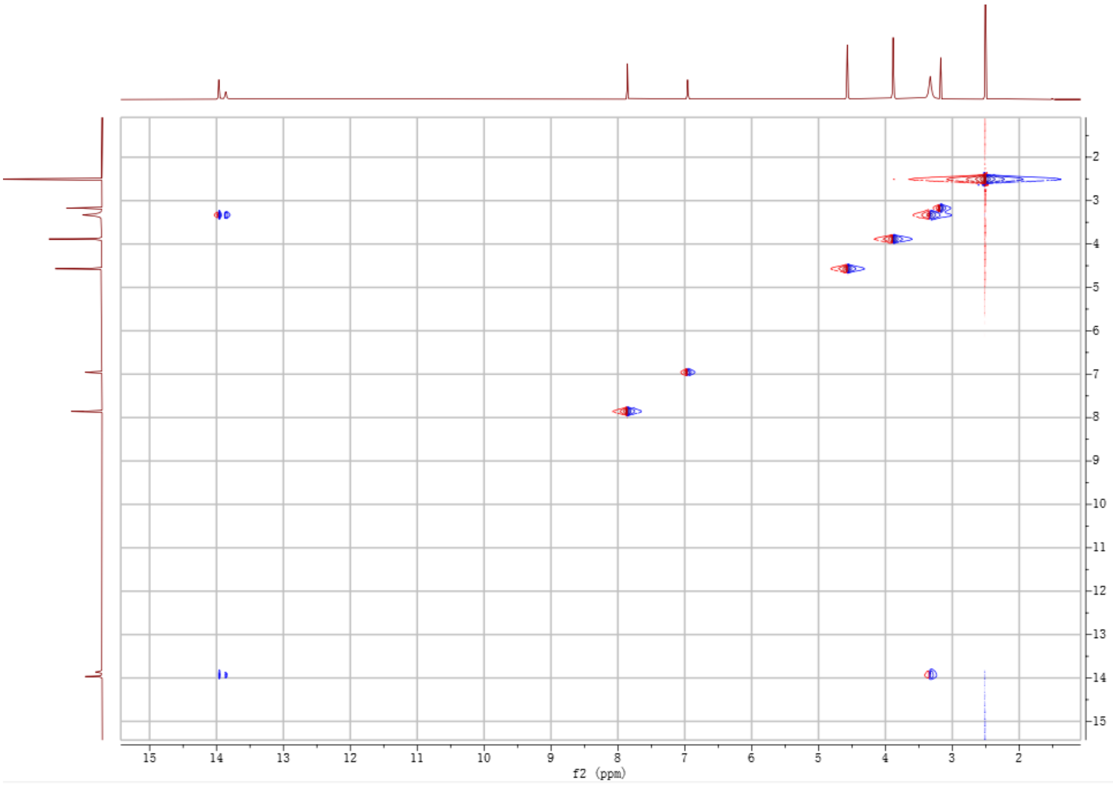

Figure S10. NOESY spectrum of compound **1** in DMSO-d_6.

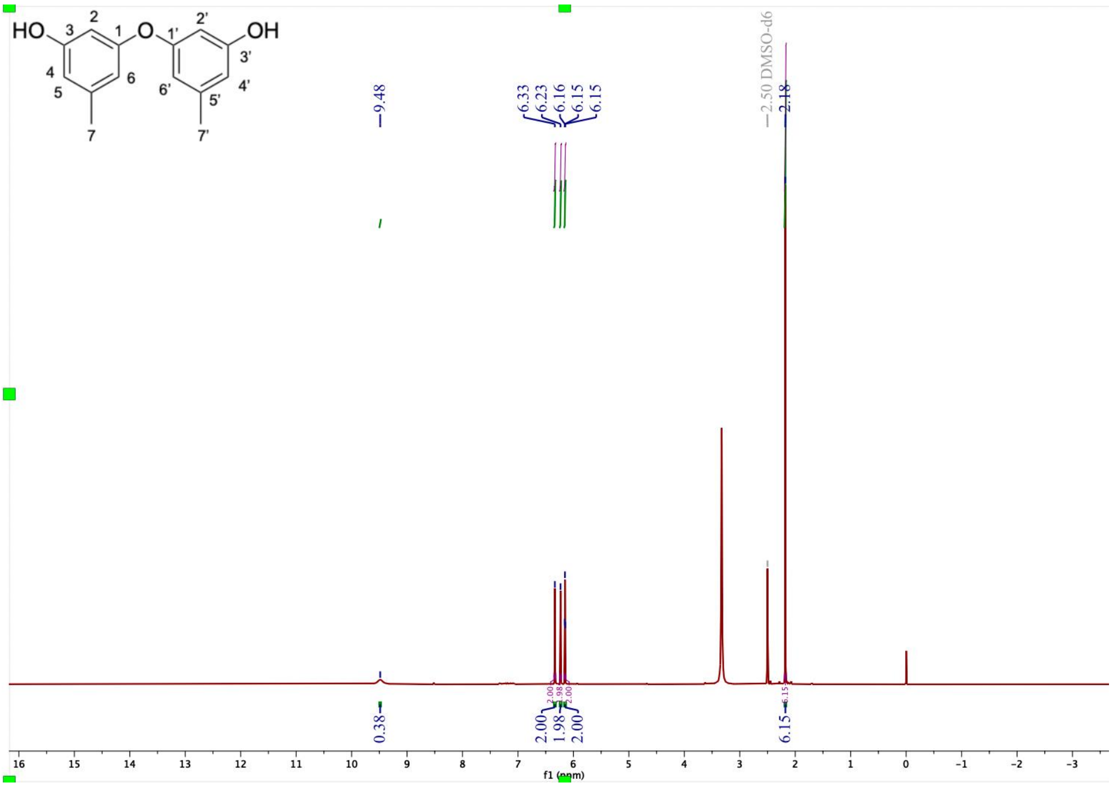

Figure S11. ^{1}H-NMR spectrum of compound **2** in DMSO-d_6 (600 MHz).

Table S2. The ^{1}H-NMR spectroscopic data for compound **2** in DMSO-d_6 (600 MHz).

Position	δ_H (*J* in Hz)
2 (2′)	6.15 (2H, *s*)
4 (4′)	6.23 (2H, *s*)
7 (7′)	2.18 (6H, *s*)
6 (6′)	6.33 (2H, *s*)

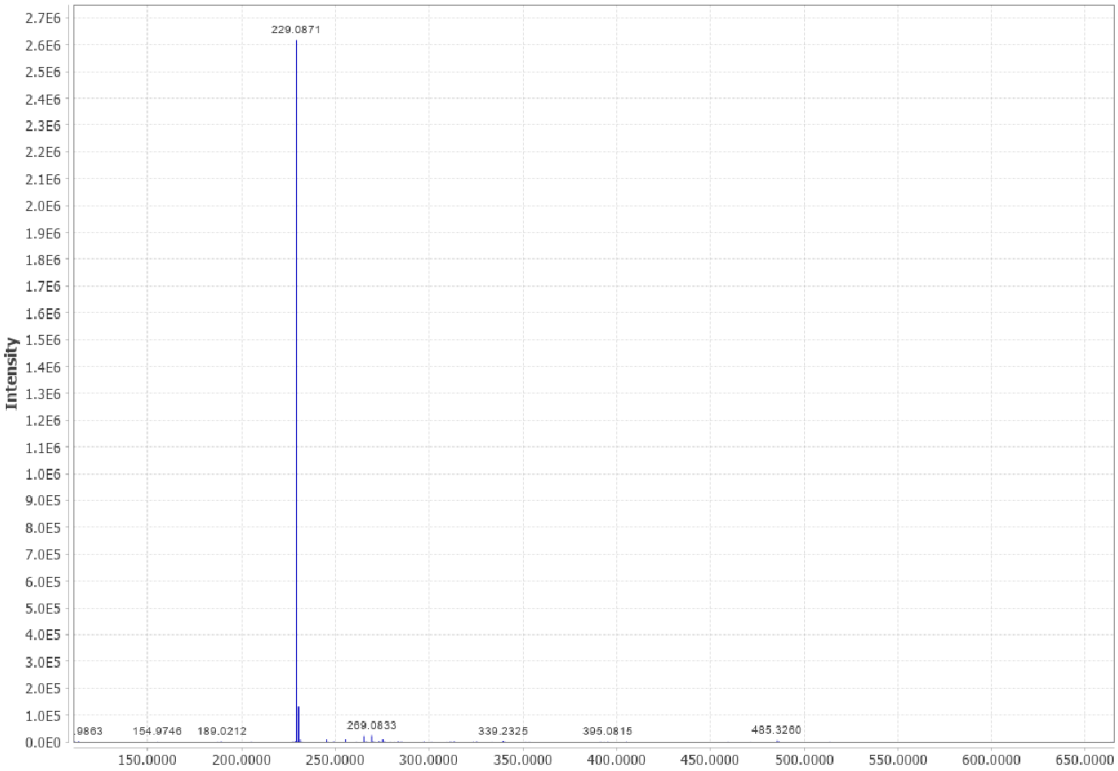

Figure S12. ESI-MS spectrum of compound **2**.

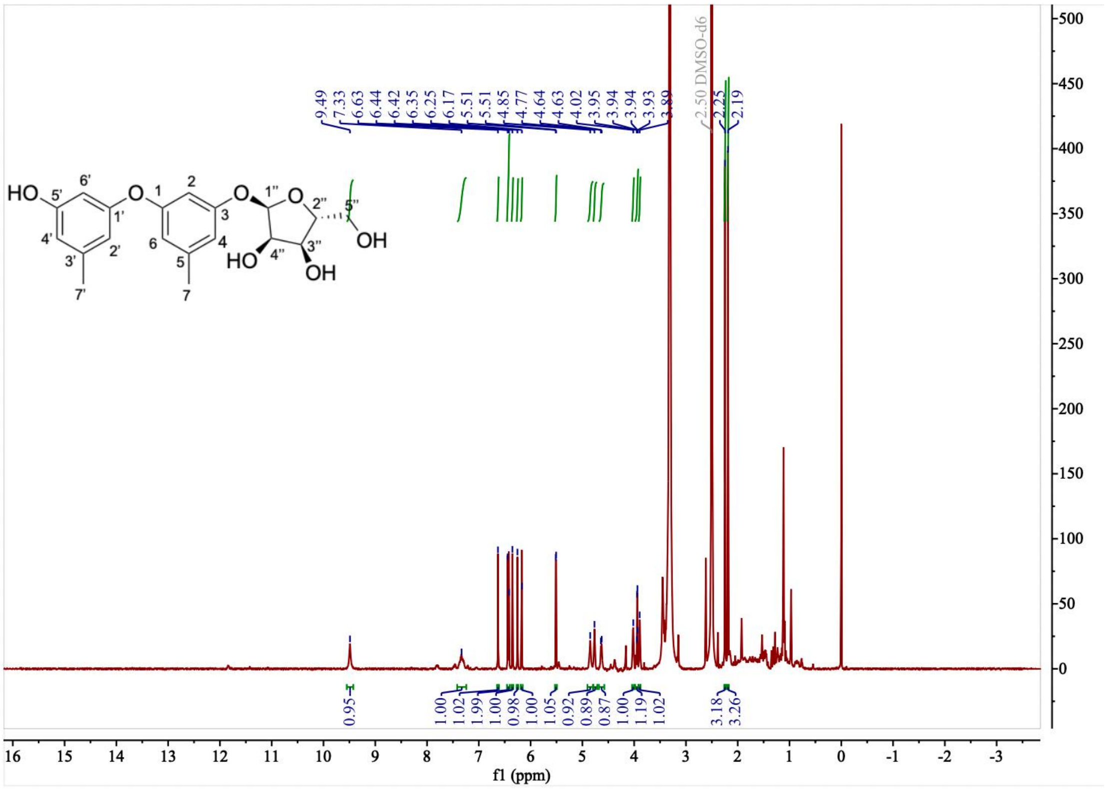

Figure S13. ^{1}H-NMR spectrum of compound **3** in DMSO-d_6 (600 MHz).

Table S3. The ^{1}H-NMR spectroscopic data for compound **3** in DMSO-d_6 (600 MHz).

Position	δ_H (*J* in Hz)	Position	δ_H (*J* in Hz)
2	6.17 (1H, *t*)	7′	2.19 (3H, *s*)
4	6.25 (1H, *s*)	1′′	5.51 (1H, *d*, *J* = 4.5)
6	6.35 (1H, *s*)	2′′	4.85 (1H, *dd*, *J* = 6.5, 4.5)
7	2.25 (3H, *s*)	3′′	4.63 (1H, *dd*, *J* = 6.5, 3.2)
2′	6.42 (1H, *t*)	4′′	4.77 (1H, *dd*, *J* = 6.9, 3.5)
4′	6.44 (1H, *s*)	5′′	3.89 (1H, *dd*, *J* = 12.1, 3.9)
6′	6.63 (1H, *s*)		3.94 (1H, *dd*, *J* = 11.7, 3.4)

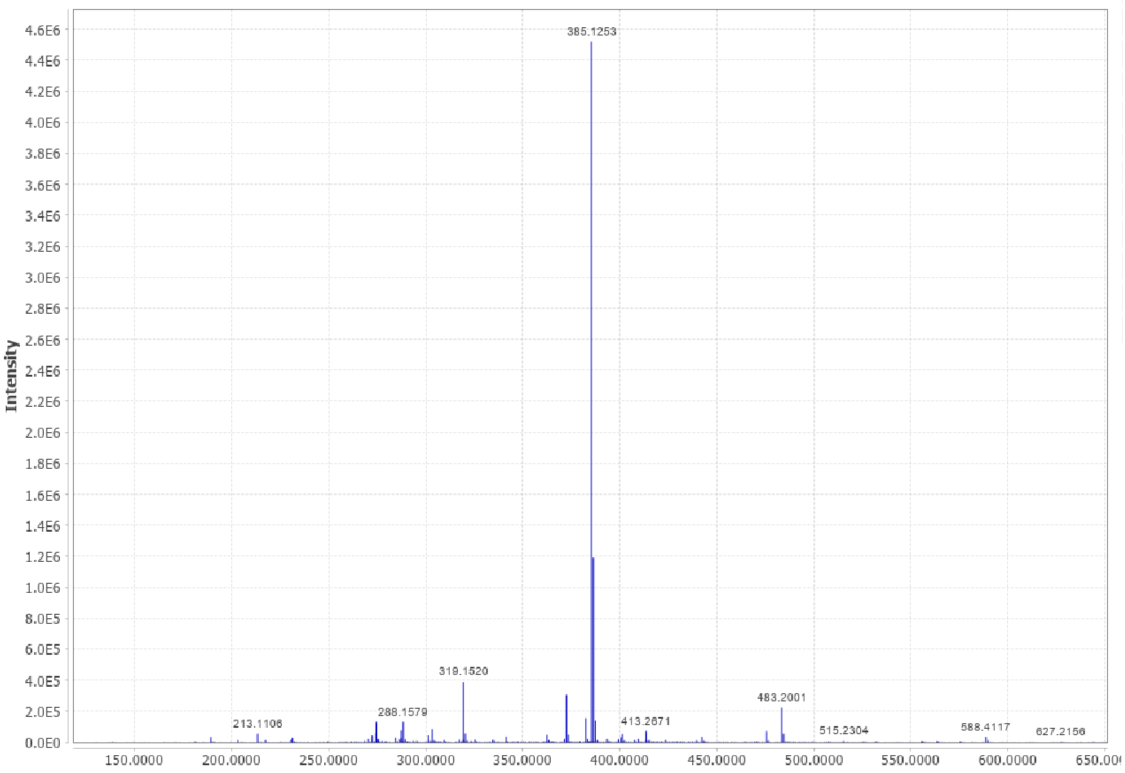

Figure S14. ESI-MS spectrum of compound **3**.

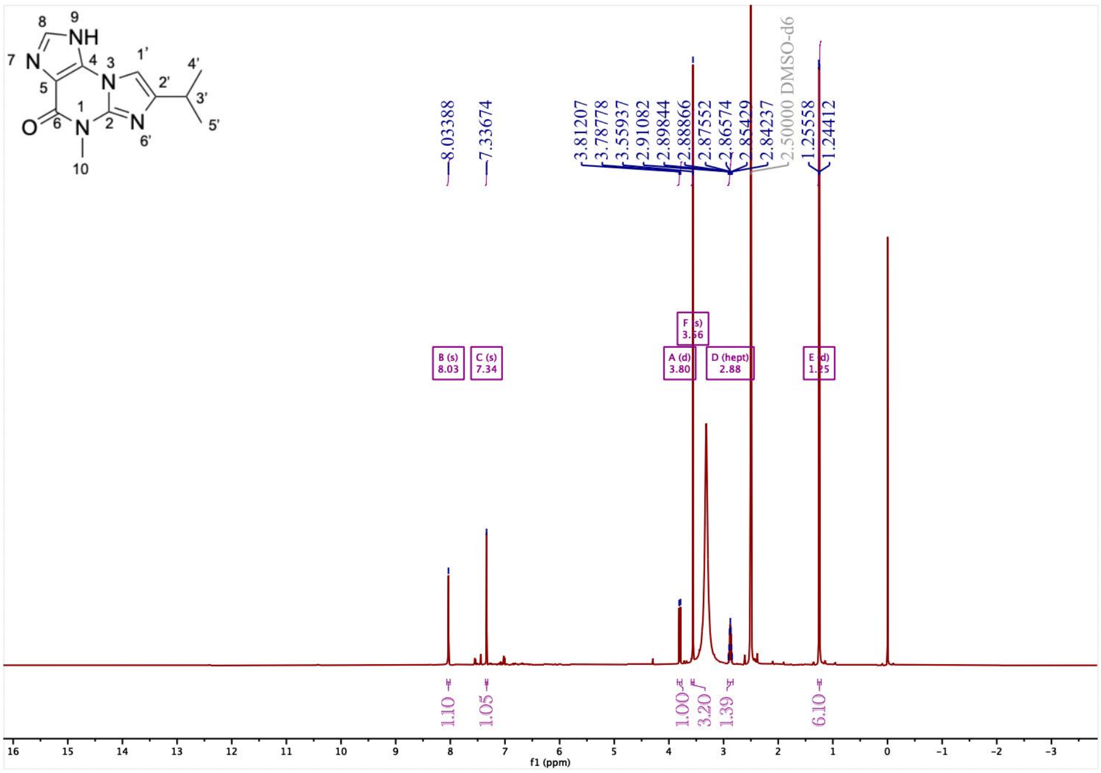

Figure S15. ^{1}H-NMR spectrum of compound **4** in DMSO-d_6 (600 MHz).

Table S4. The ^{1}H-NMR spectroscopic data for compound **4** in DMSO-d_6 (600 MHz).

Position	δ_H (*J* in Hz)
8	8.04 (1H, *s*)
10	3.57 (3H, *s*)
1′	7.34 (1H, s)
3′	2.88 (1H, *m*)
4′	1.25 (3H, *J* = 6.9)
5′	1.25 (3H, *s*, *J* = 6.9)

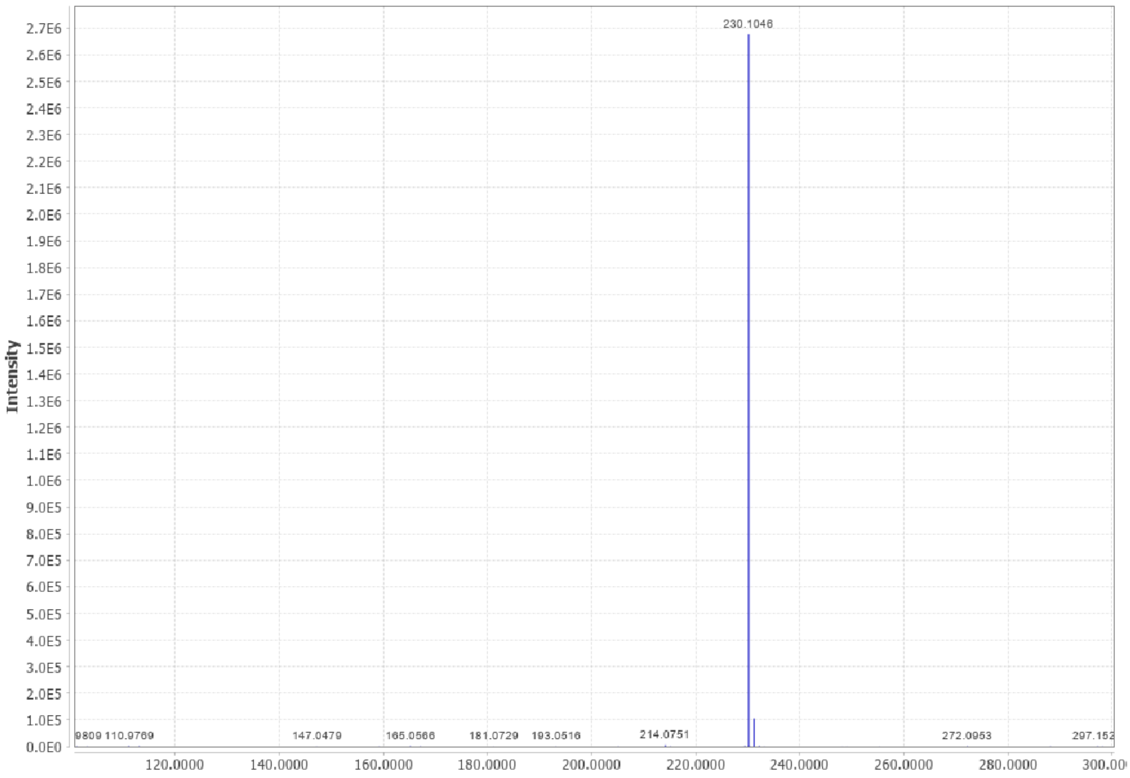

Figure S16. ESI-MS spectrum of compound **4**.

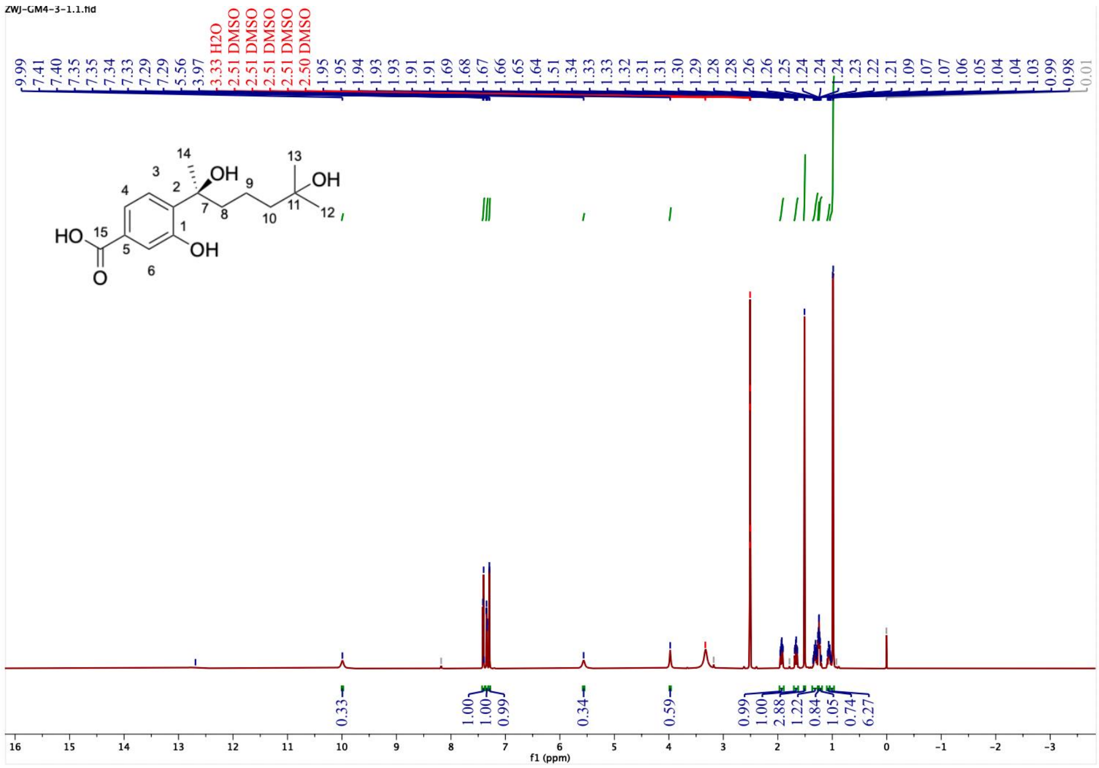

Figure S17. ^{1}H-NMR spectrum of compound **5** in DMSO-d_6 (600 MHz).

Table S5. The ^{1}H-NMR spectroscopic data for compound **5** in DMSO-d_6 (600 MHz).

Position	δ_H (*J* in Hz)
3	7.38 (1H, *d*, *J* = 8.1)
4	7.33 (1H, *dd*, *J* = 8.1, 1.7)
6	7.28 (1H, *d*, *J* = 1.7)
8	1.66-1.92 (2H, *m*)
9	1.26 (2H, *m*)
10	1.05 (2H, *m*)
12	0.98 (6H, *d*, *J* = 5.7)
13	0.98 (6H, *d*, *J* = 5.7)
14	1.50 (3H, *s*)

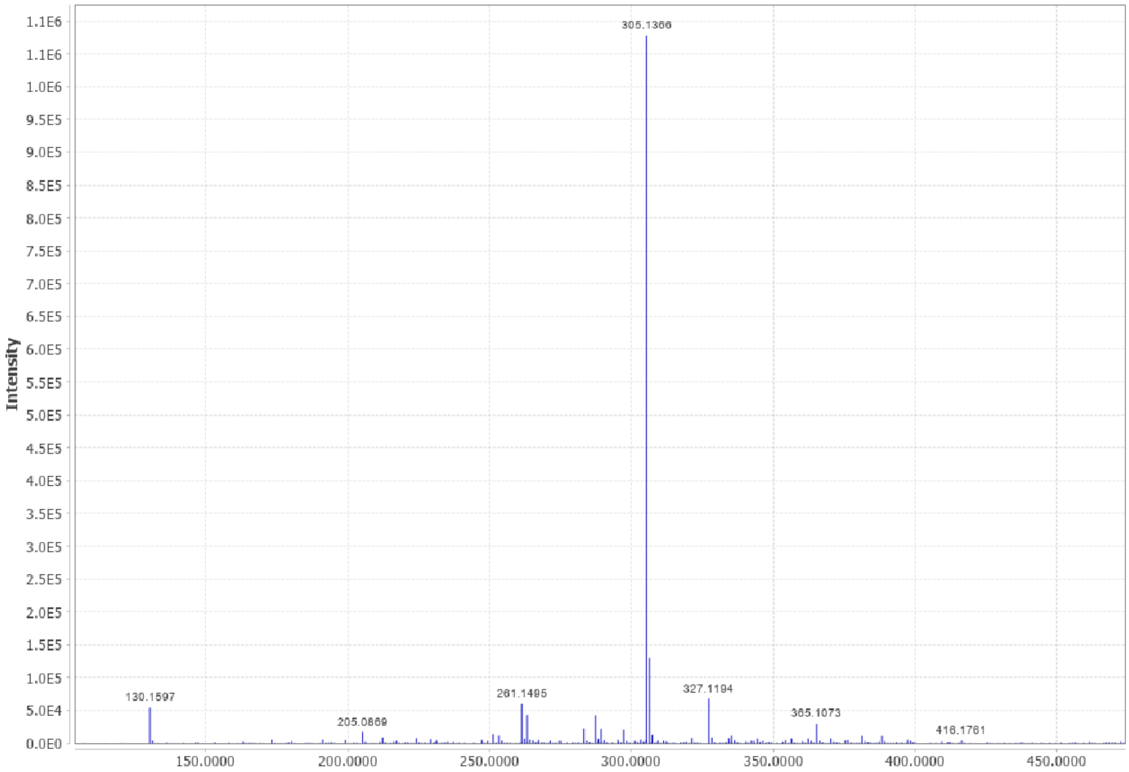

Figure S18. ESI-MS spectrum of compound **5**.

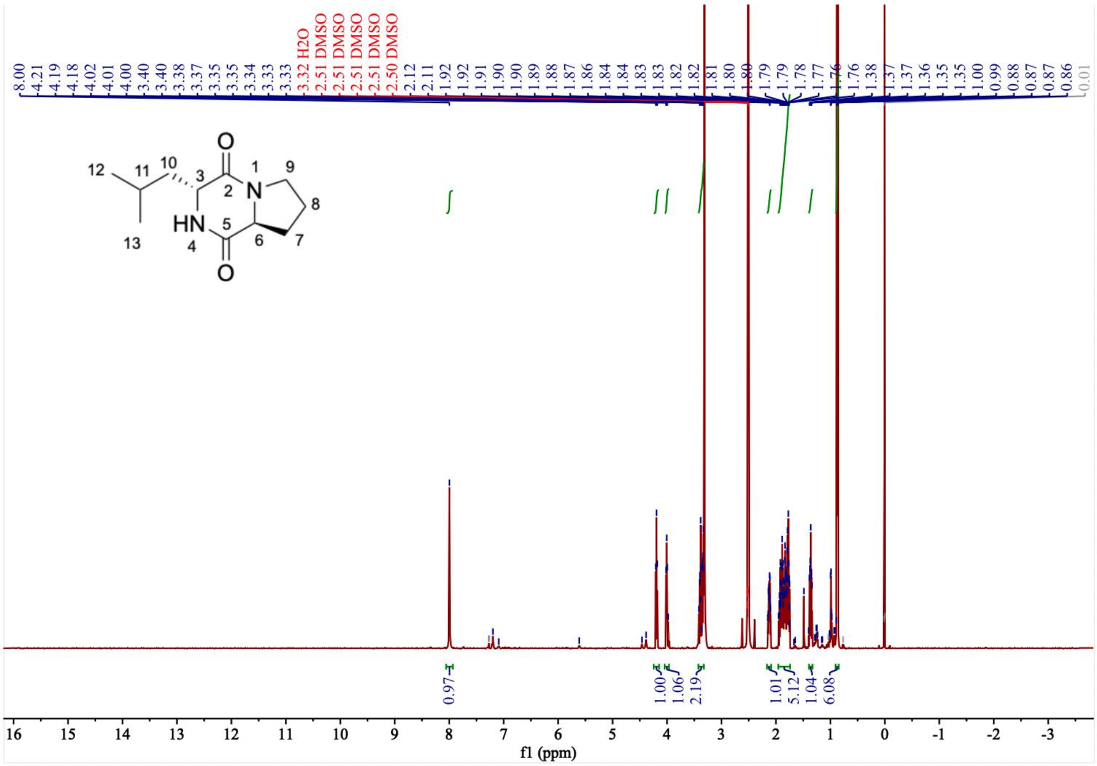

Figure S19. ^{1}H-NMR spectrum of compound **6** in DMSO-d_6 (600 MHz).

Table S6. The ^{1}H-NMR spectroscopic data for compound **6** in DMSO-d_6 (600 MHz).

Position	δ_H (*J* in Hz)
3	4.19 (1H, *t*, *J* = 8.1)
4	8.00 (1H, *s*)
6	4.01 (1H, *t*, *J* = 6.3)
7	1.83 (1H, *m*)<br>2.12 (1H, *m*)
8	1.90 (2H, *m*)
9	3.37 (2H, *m*)
10	1.77 (2H, *m*)
11	1.36 (1H, *m*)
12 (13)	0.87 (6H, *dd*, *J* = 6.6, 5.0)

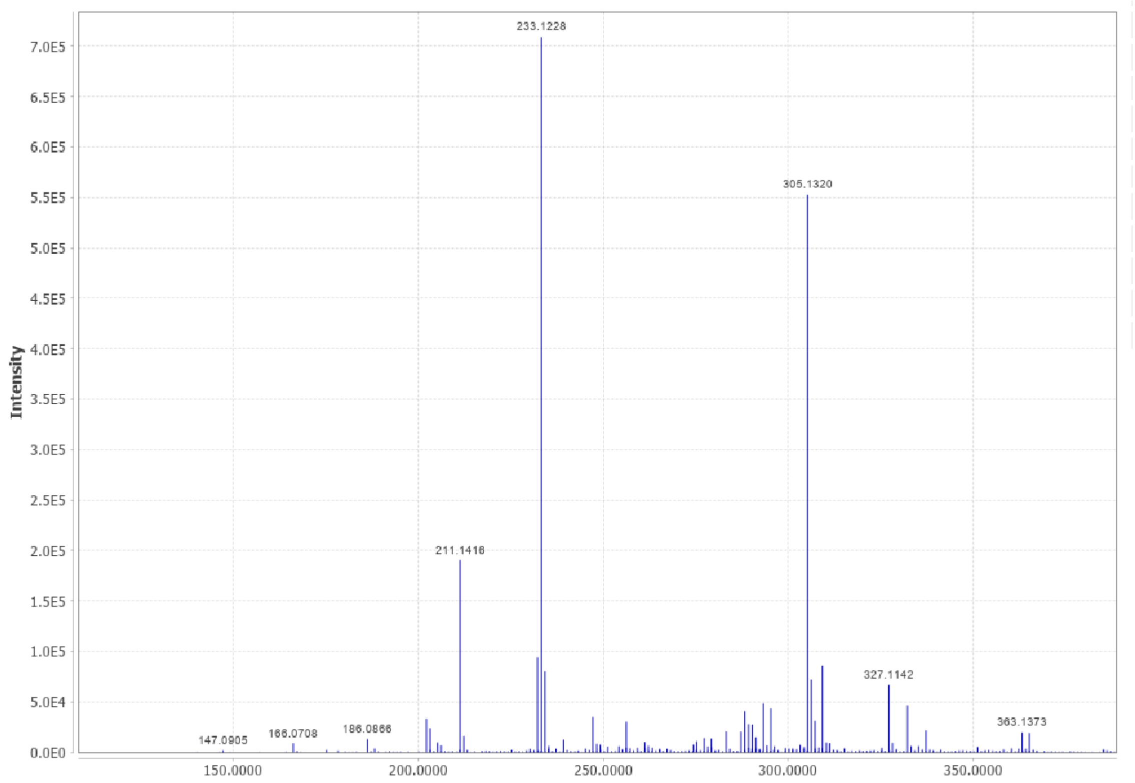

Figure S20. ESI-MS spectrum of compound **6**.

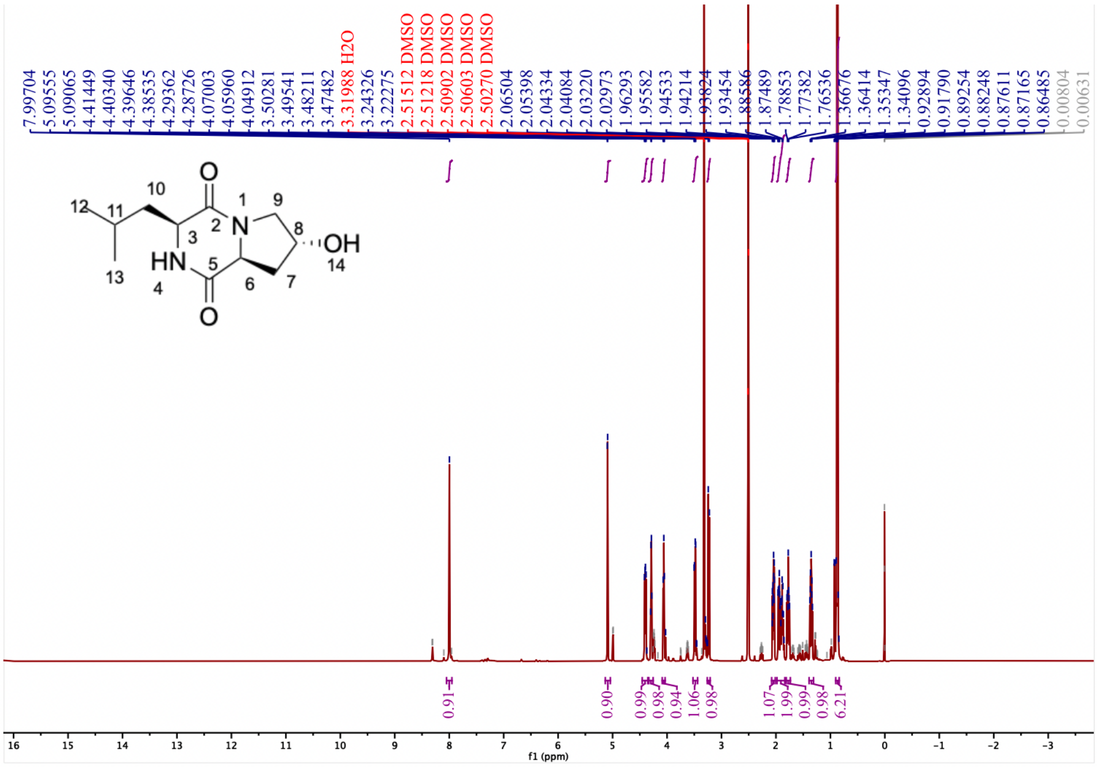

Figure S21. ^1H-NMR spectrum of compound **7** in DMSO-d_6 (600 MHz).

Table S7. The ^1H-NMR spectroscopic data for compound **7** in DMSO-d_6 (600 MHz).

Position	δ_H (*J* in Hz)
3	4.06 (1H, *m*)
4	8.0 (6H, *s*)
6	4.40 (1H, *dd*, *J* = 10.8, 6.7)
7	2.05 (1H, *m*)
	1.94 (1H, *m*)
8	4.29 (1H, *m*)
8-OH	-
9	3.49 (1H, *dd*, *J* = 12.3, 4.5)
	3.23 (1H, *d*, *J* = 12.3)
10	1.89 (1H, *m*)
	1.77 (1H, *m*)
11	1.35 (1H, *m*)
12 (13)	0.87 (6H, *d*, *J* = 6.4)

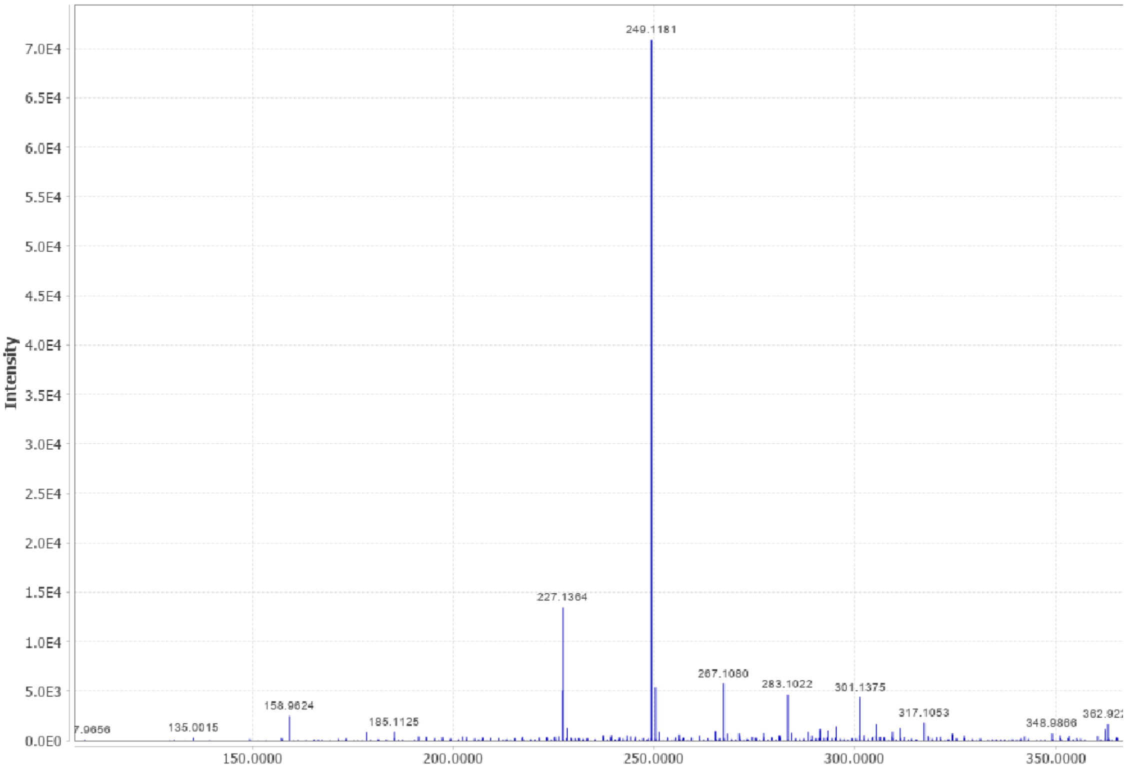

Figure S22. ESI-MS spectrum of compound **7**.

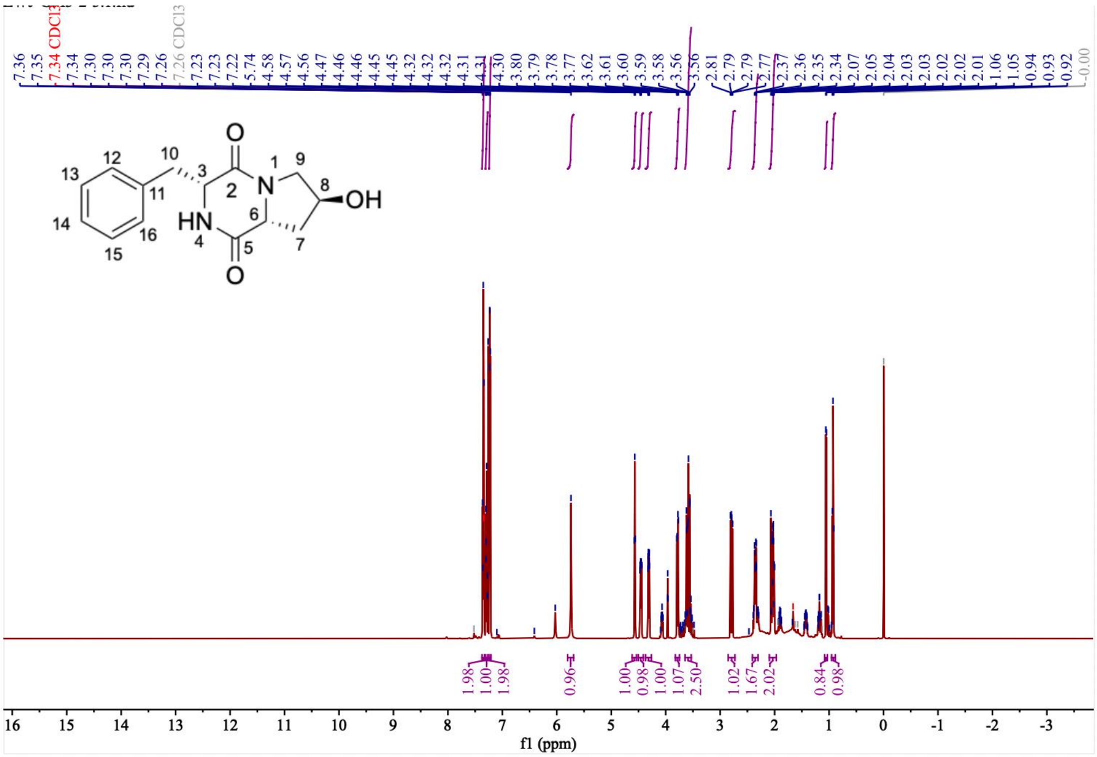

Figure S23. ^{1}H-NMR spectrum of compound **8** in CD_3OD (600 MHz).

Table S8. The ^{1}H-NMR spectroscopic data for compound **8** in DMSO-d_6 (600 MHz).

Position	δ_H (*J* in Hz)
3	4.50 (1H, *m*)
4	-
6	4.39 (1H, *m*)
7	1.40 (1H, *m*)
	2.09 (1H, *m*)
8	4.30 (1H, *t*, *J* = 4.8)
9	3.73 (1H, *m*)
	3.31 (1H, *m*)
10	3.18 (2H, *m*)
12-16	7.28 (5H, *m*)

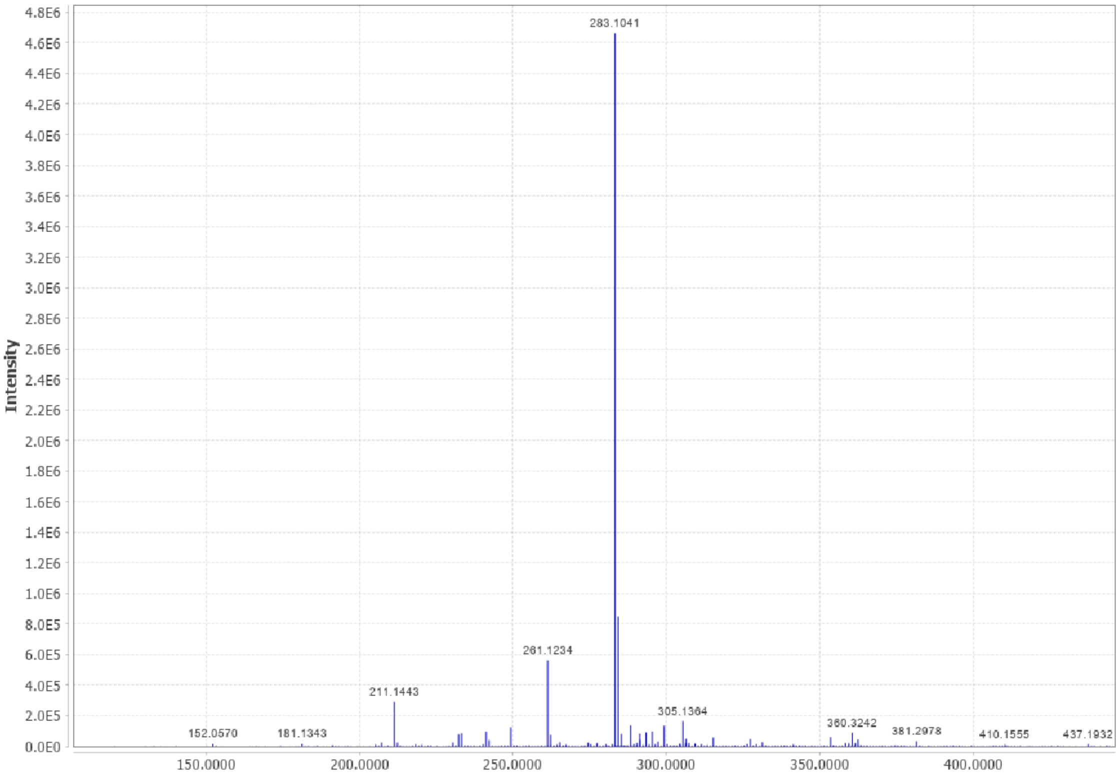

Figure S24. ESI-MS spectrum of compound **8**.

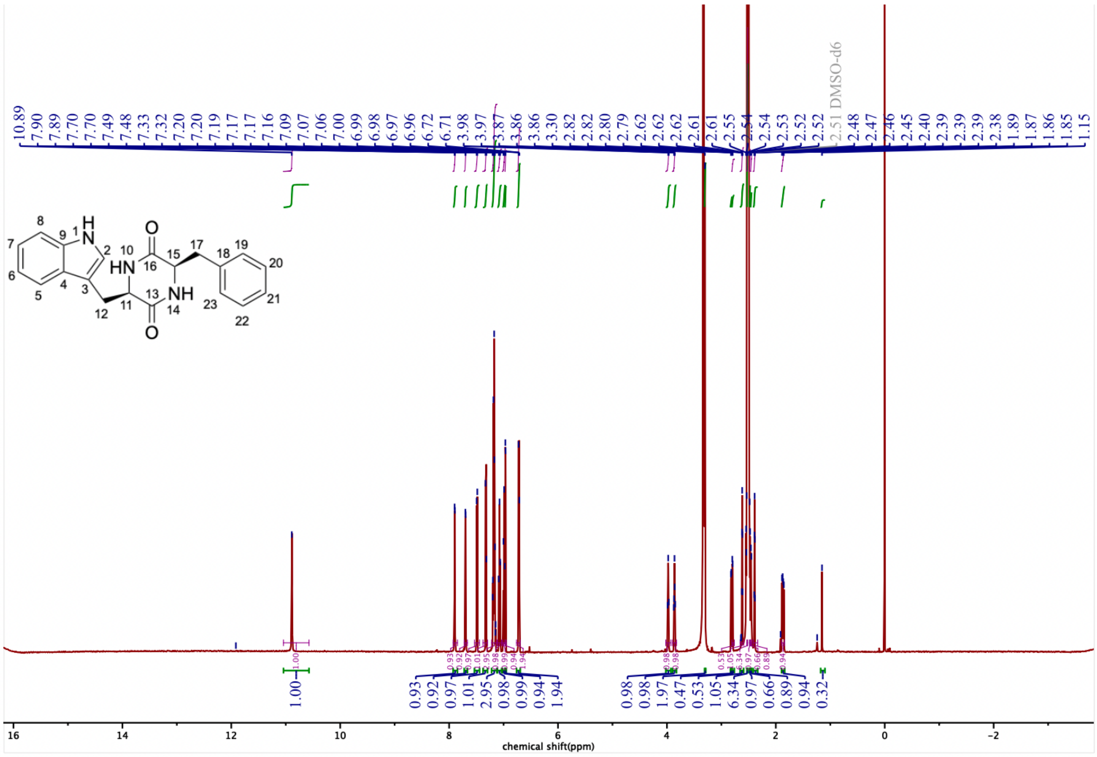

Figure S25. ^{1}H-NMR spectrum of compound **9** in DMSO-d_6 (600 MHz).

Table S9. The ^{1}H-NMR spectroscopic data for compound **9** in DMSO-d_6 (600 MHz).

Position	δ_H (*J* in Hz)	Position	δ_H (*J* in Hz)
1	10.89 (1H, *s*)	14	7.90 (1H, *d*, *J* = 2.0)
2	6.97 (3H, *s*)	15	3.85 (1H, *m*)
5	7.49 (1H, *s*)	17	2.80, 2.47 (2H, *m*)
6	6.99 (1H, *m*)	19	7.17 (2H, *d*, *J* = 7.4)
7	7.07 (1H, *t*, *J* = 7.6)	20	6.72 (2H, *d*, *J* = 6.6)
8	7.32 (1H, *d*, *J* = 8.0)	21	7.17 (1H, *m*)
10	7.70 (1H, *d*, *J* = 2.0)	22	6.72 (2H, *d*, *J* = 6.6)
11	3.98 (1H, *m*)	23	7.17 (2H, *d*, *J* = 7.4)
12	1.87, 2.62 (2H, *m*)		

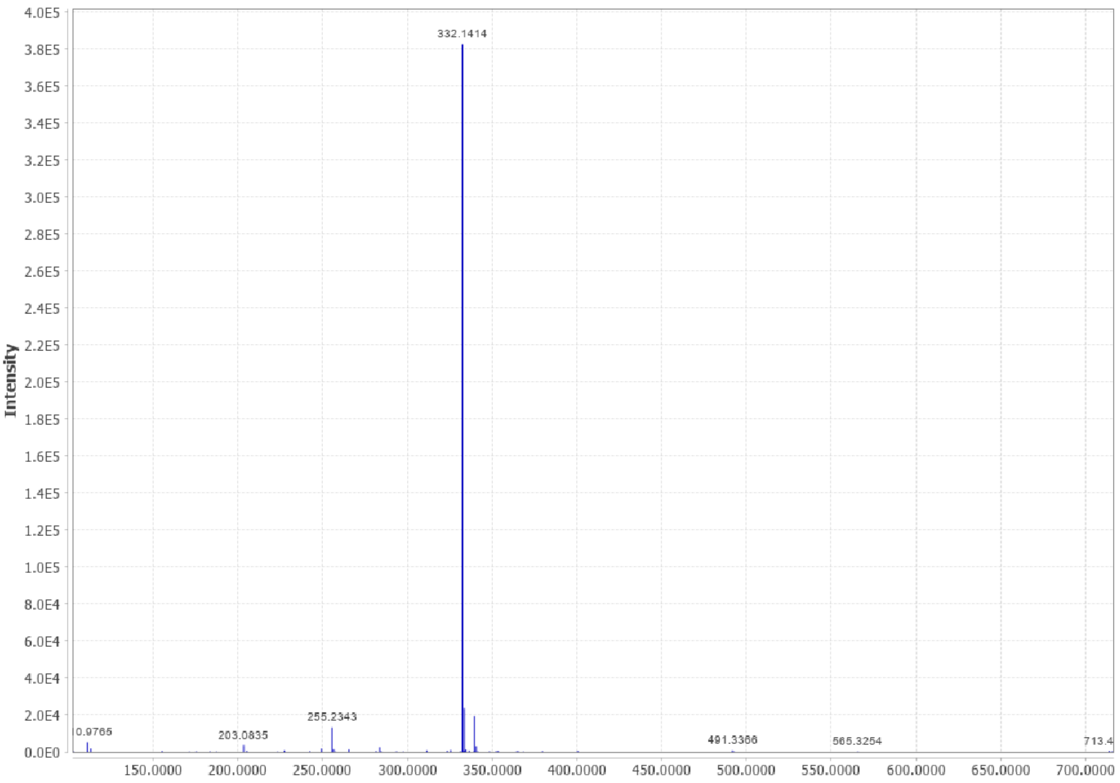

Figure S26. ESI-MS spectrum of compound **9**.

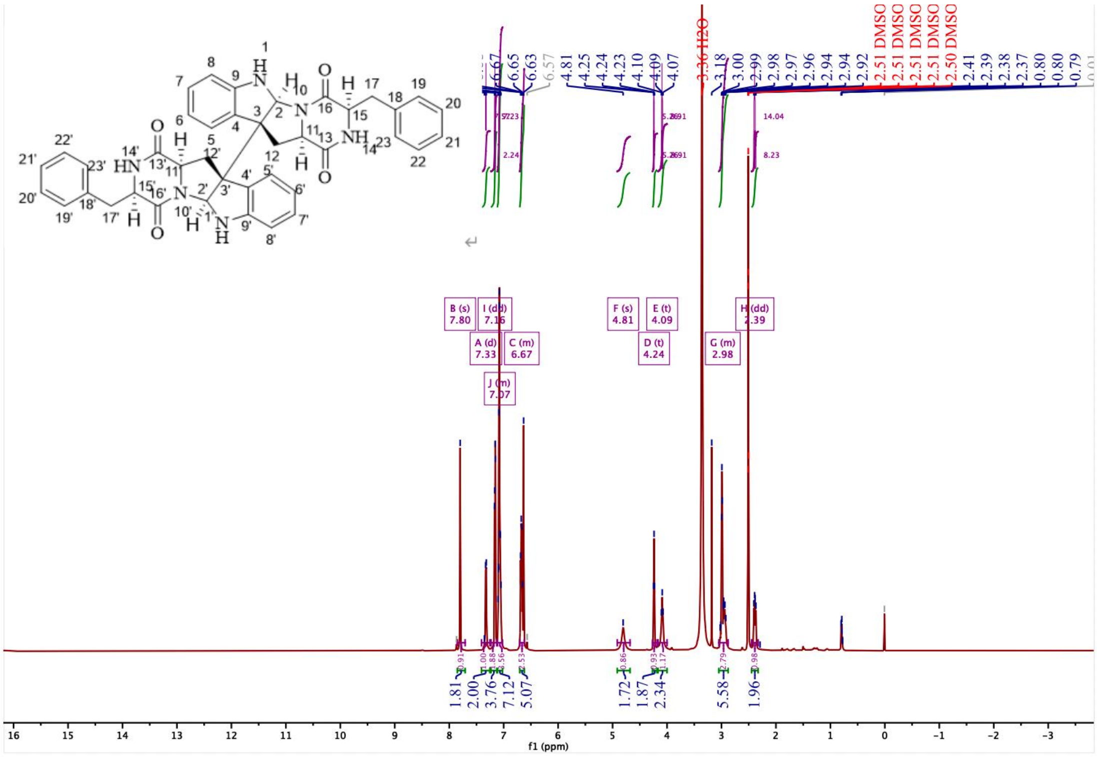

Figure S27. ^{1}H-NMR spectrum of compound **10** in DMSO-d_6 (600 MHz).

Table S10. The ^{1}H-NMR spectroscopic data for compound **10** in DMSO-d_6 (600 MHz).

Position	δ_H (*J* in Hz)	δ_C
1 (1′)	-	-
2 (2′)	4.81 (2H, *s*)	79.19
3 (3′)	-	60.11
4 (4′)	-	130.86
5 (5′)	7.33 (2H, *d*, *J* = 7.6)	125.20
6 (6′)	6.67 (4H, *m*)	118.68
7 (7′)	7.07 (8H, *m*)	129.20
8 (8′)	6.67 (4H, *m*)	109.40
9 (9′)	-	149.62
10 (10′)	-	-
11 (11′)	4.09 (2H, *t*, *J* = 8.9)	57.01
12 (12′)	2.98 (2H, *m*)	36.21
13 (13′)	-	169.38
14 (14′)	7.80 (2H, *s*)	-
15 (15′)	4.24 (2H, *t*, *J* = 5.3)	56.25
16 (16′)	-	168.48
17 (17′)	2.39 (2H, *dd*, *J* = 14.0, 8.2)	34.97
18 (18′)	-	137.50
19 (19′)	-	129.86
20 (20′)	7.07 (8H, *m*)	128.36
21 (21′)	7.07 (8H, *m*)	126.58
22 (22′)	7.07 (8H, *m*)	128.36
23 (23′)	-	129.86

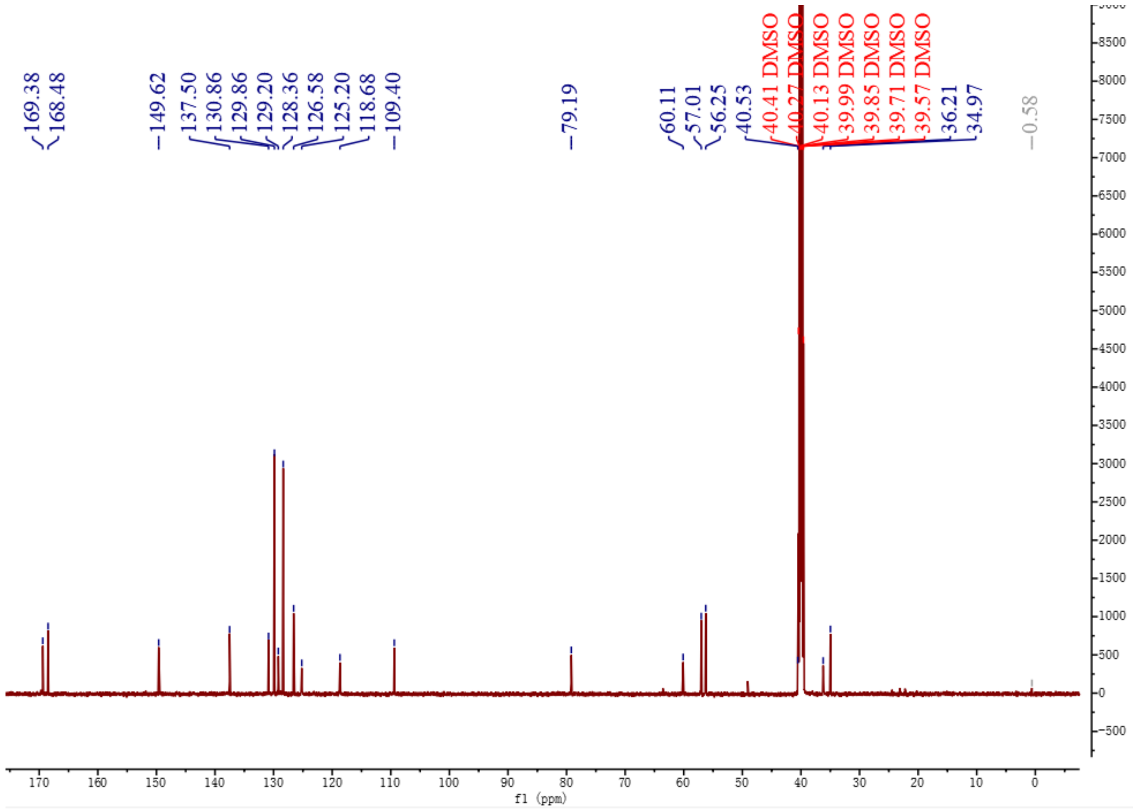

Figure S28. ^{13}C-NMR spectrum of compound **10** in DMSO-d_6 (150 MHz).

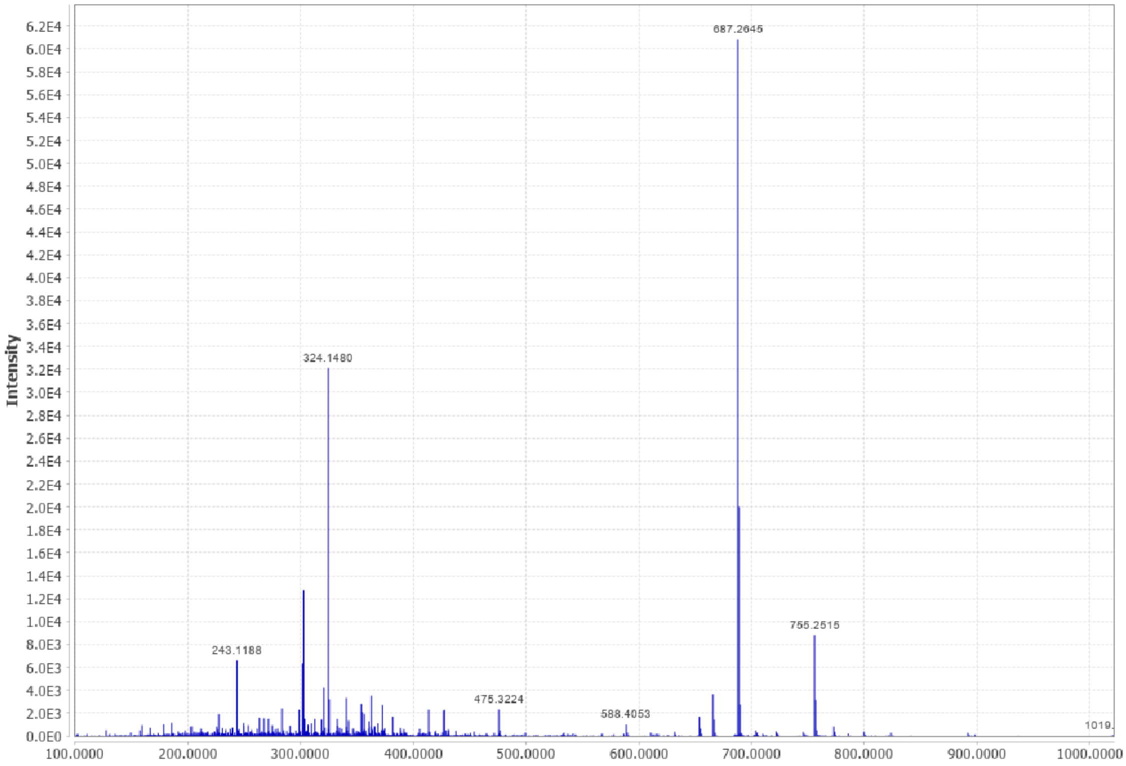

Figure S29. ESI-MS spectrum of compound **10**.
